# Machine learning-based identification of proteomic markers in colorectal cancer using UK Biobank data

**DOI:** 10.3389/fonc.2024.1505675

**Published:** 2025-01-07

**Authors:** Swarnima Kollampallath Radhakrishnan, Dipanwita Nath, Dominic Russ, Laura Bravo Merodio, Priyani Lad, Folakemi Kola Daisi, Animesh Acharjee

**Affiliations:** ^1^ College of Medicine and Health, School of Medical Sciences, Cancer and Genomic Sciences, University of Birmingham, Birmingham, United Kingdom; ^2^ Institute of Translational Medicine, University Hospitals Birmingham National Health Service (NHS) Foundation Trust, Birmingham, United Kingdom; ^3^ Centre for Health Data Research, University of Birmingham, Birmingham, United Kingdom

**Keywords:** colorectal cancer, proteins, UK Biobank, biomarkers, SHAP, translational research, machine learning, decision tree

## Abstract

Colorectal cancer is one of the leading causes of cancer-related mortality in the world. Incidence and mortality are predicted to rise globally during the next several decades. When detected early, colorectal cancer is treatable with surgery and medications. This leads to the requirement for prognostic and diagnostic biomarker development. Our study integrates machine learning models and protein network analysis to identify protein biomarkers for colorectal cancer. Our methodology leverages an extensive collection of proteome profiles from both healthy and colorectal cancer individuals. To identify a potential biomarker with high predictive ability, we used three machine learning models. To enhance the interpretability of our models, we quantify each protein’s contribution to the model’s predictions using SHapley Additive exPlanations values. Three classifiers—LASSO, XGBoost, and LightGBM were evaluated for predictive performance along with hyperparameter tuning of each model using grid search, with LASSO achieving the highest AUC of 75% in the UK Biobank dataset and the AUCs for LightGBM and XGBoost are 69.61% and 71.42%, respectively. Using SHapley Additive exPlanations values, TFF3, LCN2, and CEACAM5 were found to be key biomarkers associated with cell adhesion and inflammation. Protein quantitative trait loci analyze studies provided further evidence for the involvement of TFF1, CEACAM5, and SELE in colorectal cancer, with possible connections to the PI3K/Akt and MAPK signaling pathways. By offering insights into colorectal cancer diagnostics and targeted therapeutics, our findings set the stage for further biomarker validation.

## 1 Introduction

Globally, colorectal cancer (CRC) is the fourth most common cancer to be diagnosed and the third most common cause of death from cancer (1, 2). Each year, around 250,000 cases of CRC are identified in Europe, making up 9% of all malignancies. The incidence is slightly greater in Western and Northern Europe compared to Southern and Eastern Europe (3). Its causes are complex and multifaceted, inherited in only 5% of cases (4). Non-dietary risk factors for colorectal cancer include smoking and prolonged use of drugs like non-steroidal anti-inflammatory drugs (NSAIDs) and aspirin (5). Two screening methods are available, fecal occult blood test (FOBT) and endoscopy. Randomized studies have shown that FOBT can reduce the death rate of colorectal cancer by up to 25% in those who attend at least one screening session (6). Surgery is the primary therapy option for people with likely curable colorectal cancer. This is usually accompanied by adjuvant therapy, a systemic treatment that lowers the risk of recurrence and mortality. Additionally, pathological staging can be used to predict the recurrence rate (3). Activated oncogenes and inactivated tumor suppressor genes play crucial roles in various phases of colorectal cancer development (7). However, single-gene regulation systems cannot account for all characteristics of malignant behavior or be responsible for every cancer marker (8).

The discovery of altered proteins or metabolites during the progression of colorectal cancer is crucial in identifying novel potential biomarkers for early detection (9). Proteomics is a comprehensive and advanced methodology characterized by its ability to detect thousands of proteins simultaneously in various sample types, including cells, tissues, and bodily fluids. Research into specific biomarkers (10) and therapy pathways can greatly improve patient outcomes (11). Deciphering the underlying molecular processes and regulatory networks (12) of identified biomarkers in colorectal cancer remains an important challenge (13). For example, 5-aminovalerate interacts with the bacterial species *Adlercreutzia*, as does cholesteryl ester with *Blautia*, *Roseburia*, and *Staphylococcus.* Numerous genes have also been implicated in processes specific to epithelial cells, notably with the oxidative phosphorylation pathway and associated genes, which indirectly control cholesterol esterification in colorectal cancer (14).

Understanding the biology of cancer requires a thorough understanding of protein expression changes and interactions, which proteomics (15) provides. It has been established that proteins play a significant role in the development of biomarkers and pharmaceutical targets (16) and may be used as a window into human health (17). A comprehensive study identified 7526 proteins by label-free quantitative proteomics of 64 colon cancer tissues and 31 rectum cancer tissues are among the most comprehensive to date (18). Proteomic studies have made it easier to identify protein targets and signaling pathways involved in the development of cancer. For example, some of the proteins identified were involved in IL-17 signaling pathways in colorectal cancer progression (19). Comprehensive combined proteomic and genomic studies of CRC have been completed, leading to the discovery of treatment targets, cancer antigens, CRC subtypes, and critical signaling pathways associated with the progression of CRC (20). Even though there has been a substantial amount of research conducted on biomarkers for primary colorectal cancer, clinical guidelines in both the USA and Europe, such as those provided by the National Comprehensive Cancer Network and European Society for Medical Oncology, currently prioritize tumor-node-metastasis staging and the identification of DNA mismatch repair deficiency or microsatellite instability (21). These guidelines also consider traditional clinicopathological factors when making a diagnosis (22).

Breakthroughs in omics technologies, such as RNA sequencing (23) for transcriptome gene expression profiling, next-generation sequencing (NGS) (24), and mass spectrometry (25) have facilitated the use of molecular markers in diagnosing colorectal cancer (26). The integration of healthcare characteristics like clinical data, gene expression, and miRNA expression with machine learning and AI (27) based methods can maximize the utilization of omics data (28). Molecular interactions and biomarkers were discovered through hierarchical clustering, protein-protein networks, and correlation analyses (29). Proteomic signatures, which offer a tool for forecasting CRC stages and identifying biomarkers, were constructed by selecting differentially expressed proteins (DEPs) using Least Absolute Shrinkage and Selection Operator (LASSO) and Support vector machine (SVM) (30). Sensitive diagnostic tools to detect cancer were found using machine learning algorithms like SVM, random forest, and decision trees (31). Other non-omics methods have been used such as nanotechnology (32) and imaging techniques to better understand the disease (33). Despite the advancements made in understanding the molecular characteristics, biological markers, and therapeutic targets of colorectal cancer, the complexity of its biology, severe outcomes, and extensive metastasis highlights the need for additional research in identifying predictive and prognostic biomarkers (34).

Here, we present the comprehensive analysis of the Olink-based quantitative proteomics in UK Biobank data. With the application of Explainable artificial intelligence (XAI) models and validation of models with the *Bosch* et al. dataset, our work aims to identify protein biomarkers and their mechanisms that may be employed as diagnostic markers across a range of proteomic datasets. Ultimately, these discoveries might lead to improved cancer detection and the emergence of greater accuracy models.

## 2 Materials and methods

### 2.1 Study design and participants

The study was carried out in two phases, initially using proteomics data sourced from UK Biobank, with the cohort in *Bosch* et al. as a validation dataset. The UK Biobank is a large-scale cohort research project, consisting of 500,000 individuals recruited between 2006 and 2010 from various sites around the UK, aged 40 to 69.

Colorectal cancer patients were first identified using diagnosis information from various sources of UK Biobank, with all details and diagnosis codes detailed in **Supplementary Table S1**. Then participants with age and sex were matched to participants with no ICD-10 diagnosis in their inpatient data using the MatchIt package. Participants with metabolite information (as of July 2021) and proteomic data (as of July 2023) were followed up for analysis.

**Figure 1:**
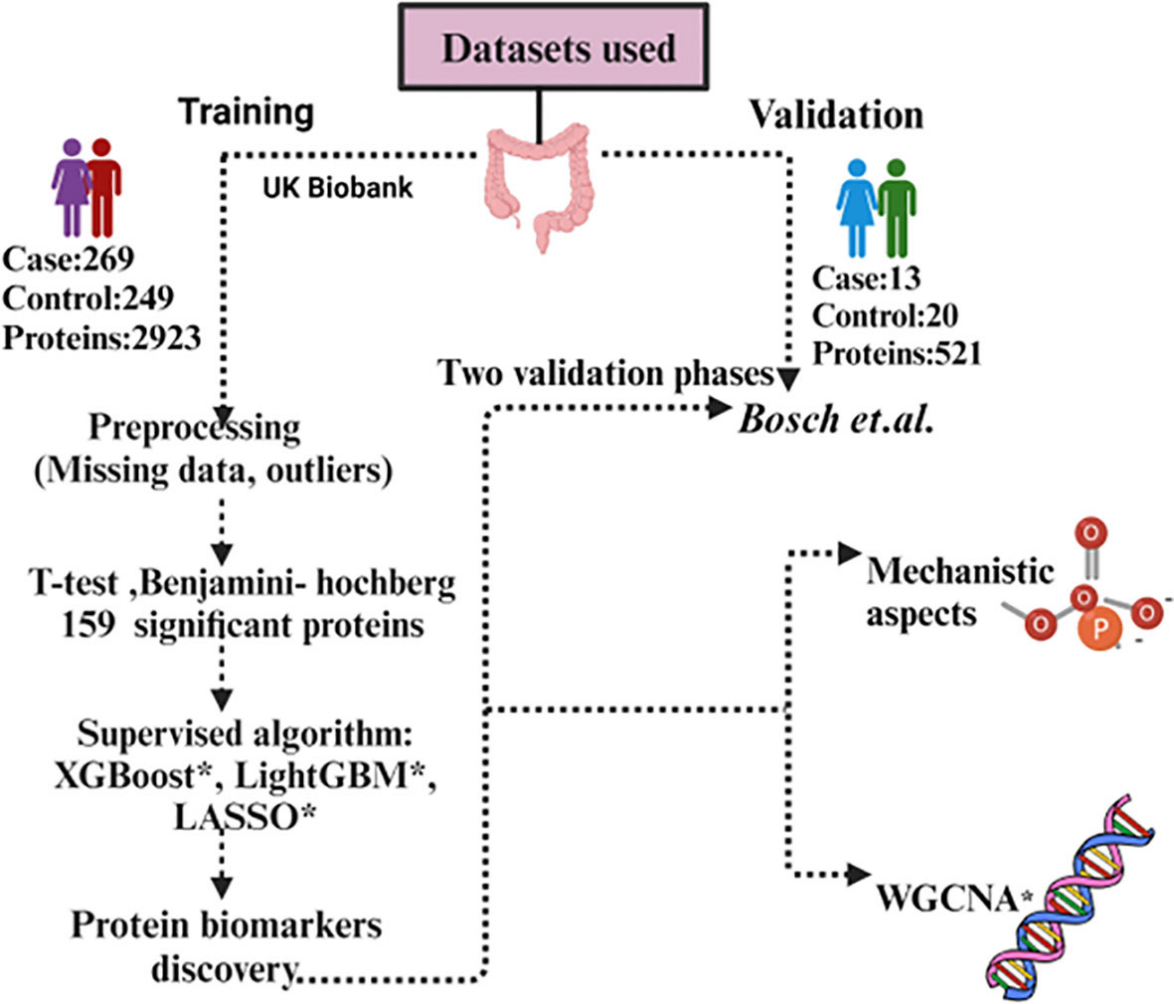
Workflow of the data analysis using UK Biobank and *Bosch* et al. dataset. *XGBoost, eXtreme Gradient Boosting; *LASSO, Least Absolute Shrinkage and Selection Operator; *LightGBM, Light Gradient-Boosting; *WGCNA, Weighted gene co-expression network analysis.

We conducted our comprehensive proteomics data analysis using two datasets which include one from the UK Biobank and another from *Bosch* et al. UK Biobank is used as training and *Bosch* et al. used as a validation dataset. The details of the datasets used in this study are summarized in **Table 1**. In the first phase,159 significant proteins of UK Biobank were used to validate with *Bosch* et al. data, and in the second phase we used 98 proteins that were common among both datasets to validate (**Figure 1**). For UK Biobank, proteomics data was generated using the antibody-based Olink Explore 3072 PEA (35) and for *Bosch* et al. (36) proteins were generated using liquid chromatography-mass spectrometry (LC-MS).

**Table 1:** Datasets used in the study.

Proteomics Dataset	Technology/platform	Number of samples, Proteins
UK Biobank (Training)	Olink	Case=269 Control=240 Proteins=2923
*Bosch et.al.* (Validation)	LC-MS/MS *	Case=13 Control=20 Proteins=521

*LC-MS/MS, Liquid Chromatography-Mass Spectrometry/Mass Spectrometry.

### 2.2 UK Biobank data processing

All pre-processing steps are performed using R (v4.2.0). Imputation of 20% missing value protein which has 1460 proteins (37) was done.

We used several methods mean, median, Classification and regression trees (CART), and K-Nearest Neighbors (KNN) for imputation to compare the distribution before and after imputation (**Supplementary Figures S1A–E**). The mean imputed dataset had a close relation to the original dataset distribution. Kolmogorov Smirnov Test (K-S test) is also performed to find the statistical difference among different imputation methods. Mahalanobis distance (38) has been used for outlier detection based on the 99^th^ percentile threshold using the mean and covariance matrix of our data. It was determined that the dataset’s threshold was 22.71. Six samples were found to be outliers (**Supplementary Figure S2**) and discarded from the study. For further statistical analysis and machine learning tasks, the pre-processed dataset (1460 proteins and 512 samples) was used.

### 2.3 Machine learning model

In this study, we used three machine learning models, eXtreme Gradient Boosting (XGBoost) (39). Light Gradient-Boosting Machine (LightGBM) (40), and Least Absolute Shrinkage and Selection Operator (LASSO). XGBoost and LightGBM make use of an ensemble of classification trees and combine their prediction from multiple individual decision trees to make more accurate and robust predictions, LASSO on the other hand, it is a regularization algorithm that helps reduce the feature space, highlighting key association. All machine learning analysis were carried out in python (v3.9.13) “scikit learn” module (v1.0.2).

For this study, the selection of LASSO, XGBoost, and LightGBM is based on several aspects. With strong feature selection and regularization capabilities, LASSO regression is especially well-suited for high-dimensional datasets, which are frequently employed in biomedical research. By implementing a penalty, LASSO reduces overfitting and efficiently selects the most pertinent predictors by shrinking less significant coefficients to zero (41). Research has shown that LASSO is useful for finding important risk variables and biomarkers in cancer studies. The interpretability and accuracy of predictive models were enhanced when Ma et al. (42) effectively used LASSO to choose prognostic characteristics in colorectal cancer. XGBoost is well-known for its high Area Under the Curve (AUC) scores in classification tasks, its ability to handle missing data and non-linear interactions (39), and its successful application in predicting the survival of colorectal cancer (43). LightGBM is perfect for large-scale datasets because it is scalable and fast, manages high-dimensional, sparse data effectively, and provides superior AUC (40). Recent research, including Zhang et al. (44) and Onwuka et al. (27), supports the application of these models by showing that they may accurately and clinically meaningfully identify the relationships between variables in colorectal cancer. The study utilized SHapley Additive exPlanations (SHAP) to enhance the interpretability of the machine learning models, addressing a key limitation of complex algorithms like XGBoost and LightGBM—their “black-box” nature. Even though these models are quite effective and precise, it is important to comprehend their predictions in a clinically useful way, particularly in colorectal cancer research where it is critical to identify important risk variables and how they affect the result. By quantifying each feature’s contribution to the model’s output, SHAP offers a cohesive framework for explaining individual predictions (45). By using SHAP, the study makes sure that the high AUC scores achieved by the machine learning models are accompanied by interpretability and transparency, which makes the results useful for clinical decision-making. The study’s objective of identifying important risk variables and their impact on the outcomes of colorectal cancer is also in line with SHAP’s ability to identify feature relevance at both the global (model-wide) and local (individual prediction) levels. Each of the methods has complementary properties. LASSO is a linear method and able to perform sparse feature selection, by shrinking the coefficients of less important features to exactly zero and hence captures the most comfortable linear relationships. XGBoost and LightGBM, however, are more versatile, and capable of capturing complex, non-linear relationships in the data. LightGBM is able to capture model building for categorical features. Together with SHAP for interpretability, these three models—LASSO, XGBoost, and LightGBM—complement one another by fusing their specific strengths to provide a robust and clear analysis. LASSO helps by regularizing the model, decreasing overfitting, and enhancing interpretability by efficiently managing high-dimensional data. This aids in the model’s simplification, making it easier to handle and understand. In contrast, XGBoost (39) and LightGBM) (40), are strong boosting algorithms that offer high predictive accuracy, both of which are important for producing accurate predictions. SHAP depicts how each feature affects the model’s predictions to give an understanding of feature relevance.

### 2.4 Hyperparameter optimization

The proteomic dataset underwent an initial 80%-20% train and test split (27). We employed the GridSearchCV (46) method to determine the most effective values for each model’s hyperparameters. GridSearchCV is a utility within the Scikit-learn library designed for iterating through predetermined hyperparameters and training the model on the provided dataset. Stratified k-fold cross-validation with 5-fold was employed within grid search ensuring robust model assessment and hyper-parameter tuning by assessing all possible combinations of specified parameter values. The accuracy metrics collected for each hyperparameter combination were used to decide on top-performing models.

### 2.5 Machine learning model evaluation

The study utilized the UK Biobank dataset for training and testing and the data from *Bosch* et al. for validation of the performance of three distinct machine learning models (LASSO, LightGBM, and XGBoost). Each model underwent evaluation using various metrics including precision, recall (47), specificity (48), F1 score, and AUC-ROC (49). We incorporated a validation strategy to assess the quality of our findings and test the robustness of the proteins found in our model. Our experimental methodology utilized the k-fold cross-validation technique (k=5), a widely accepted approach (50). The model’s effectiveness was assessed by calculating the average performance metrics across 50 iterations (**Supplementary Figure S3**).

### 2.6 Feature selection

We used SHapley Additive exPlanations (SHAP) for feature selection. SHAP quantifies the significance of each characteristic by utilizing ideas from cooperative game theory to explain how each protein contributes to the model’s predictions. This method improves the model’s interpretive skills while also offering insights into the decision-making process, which raises the output of the model’s transparency (51). This was visualized using a SHAP global importance plot to identify the proteins that had the greatest influence. Additionally, plots were used to show the relative contributions of each top protein to the sample. Finally, the common proteins of both datasets were visualized using a heatmap.

### 2.7 Statistical analyses

The present study used R (v4.3.0) for all pre-processing analyses of the UK Biobank, while Python (v3.9.13) was used for machine learning and interpretable XAI-based modeling. The Benjamini-Hochberg principle (52) was used to adjust p-values for multiple testing corrections, with values that fell below the critical false discovery rate (FDR) of 0.05 deemed significant. When dividing the datasets between sets for testing, training, and cross-fold validation for the machine learning tasks, they were stratified according to the target class. The mean AUC score across the folds together with the 95% confidence interval in Python provided a representation of the models.

### 2.8 Knowledge integration using STRING database

The protein–protein association network is one of the most efficient, wide-ranging forms of networks, it includes every gene that codes for a protein in a particular genome and depicts the functional interactions between those genes (53). Protein-protein interactions that are known or predicted are documented in the STRING (54) database. We compiled the list of proteins from both phases and uploaded it to STRING for analysis with a confidence interval of 0.4 classified as ‘medium confidence’ for predicted interactions and obtained a comprehensive view of potential interactions.

### 2.9 Human protein atlas

To understand the dynamic expression of protein-coding genes and to generate a map of the human proteome, the Human Protein Atlas (HPA) project was initiated as a part of the Human Proteome Project (HPP) focusing on antibody-based proteomics and integrated omics (55). The objective of HPA is to identify the expression and spatial distribution of each human protein in various human organs, cancer types, and cell lines. We have employed HPA to get more knowledge of the significant role that proteins play in the many types of cancer cells.

### 2.10 Weighted gene co-expression network analysis

We performed a Weighted Gene Co-expression Network Analysis (WGCNA) (56) on both of our datasets to identify modules of co-expressed genes and their association with clinical traits, specifically focusing on cancer and non-cancer control samples. The analysis began with the selection of an optimal soft-thresholding power (β) to ensure the network’s scale-free topology.

Next, a module detection test was carried out via the hierarchical clustering method to identify distinct modules with co-expressed genes. After identifying distinct modules, we performed a correlation matrix with cancerous and non-cancerous samples to indicate genes that are upregulated or downregulated in diseased cases. The list of upregulated or downregulated genes was assessed for functional annotations from the Kyoto Encyclopedia of Genes and Genomes (KEGG) via pathway enrichment analysis to identify biological pathways that can serve as potential therapeutic targets.

### 2.11 Protein quantitative trait loci analysis

Proteomic analysis was carried out for seven proteins (AHCY, CEACAM5, LCN2, RETN, SELE, TFF1, and TFF3) in the UK Biobank proteomics data against imputed SNP array data (57). The cohort was split into controls without CRC, those who had CRC before the blood sample was taken, and those who received a diagnosis afterward (numbers depend on missingness of proteomics data but a maximum of 51002, 332, and 884 respectively). Analysis was carried out using regenie (58) v3.4.1, with covariates including sex, age, age^2^, age x sex, age^2^ x sex, genetic principal components 1-10, and genetic array used. Sex was coded as 1 for females and 2 for males. Gene annotations were applied using biomaRt (59) v2.56.1, with the dataset hsapiens_gene_ensembl for GRCh37.

### 2.12 Batch-effect correction

The common proteins between the UK Biobank and *Bosch* et al. datasets were combined for ComBat analysis (60). Batch effects (61) are expected to have an impact on the integrated dataset due to the variations in cohort characteristics and data-collecting techniques. Using the R package “sva,” (62) we implemented the ComBat batch-effect correction method to resolve these disparities. This method adjusted for systematic differences while maintaining significant biological diversity, harmonizing the protein expression data across datasets. This stage made sure that the source of the dataset wouldn’t affect subsequent analysis. After batch-effect correction, batch corrected dataset was split for testing (20%) and training (80%). We used a 5-fold cross-validation technique in the training phase to ensure a reliable and unbiased assessment of predicted performance. Three models for prediction were used LASSO regression, LightGBM, and XGBoost. To optimize performance, hyperparameter optimization was done using grid search. Each model’s performance metrics, such as accuracy, ROC-AUC, precision, recall, and F1-score, were computed in order to thoroughly assess how well it predicted the variable being studied. Further we analyzed the potential biomarkers that were found to be particularly important to the desired outcome, in addition to assessing the models on the entire dataset, and the same performance measures were used to evaluate each model’s predictive value. This provided us with information about the relative significance and predictive ability of these particular proteins by comparing the performance of the entire dataset with those on individual biomarkers. To evaluate feature importance and determine which feature had the most influence on model predictions, potential proteins were then put through SHAP analyses. A better comprehension of the variables influencing the models’ outputs and their conformity to biological relevance was made possible by the results of this study.

## 3 Results

### 3.1 Dataset cohort

After extracting participants in UK Biobank with a colorectal diagnosis (as defined in **Supplementary Table S1**), 9,890 participants were identified, with 6372 having a colorectal diagnosis coming from more than 1 source and 3518 coming from just a unique source. From those unique sources, death registries identified 74 participants, cancer registries 325, hospital inpatient data 2737, and self-reported information 382. These 9,890 participants were then age and sex-matched to controls (as defined by having no ICD-10 diagnosis recorded in hospital inpatient data) generating a dataset of 19784 participants. After filtering for those with metabolite information (as of July 2021) and protein data (as of July 2023), 509 participants had all information remaining, corresponding to 269 cancer patients and 240 controls. 5 participants had extra protein follow-up information, yielding a total of 518 samples with protein information used. Basic demographic information can be found in **Table 2**.

**Table 2:** Baseline demographic characteristics.

Characteristics	UK Biobank dataset			*Bosch et.al.*	
	Total (N=509)	Colorectal cancer (N=269)	Control (N=240)	Colorectal cancer (13)	Control (20)
Sex					
Male	290 (57.0%)	163 (60.6%)	127 (52.9%)	6 (50)	14 (70)
Female	219 (43.0%)	106 (39.4%)	113 (47.1%)	–	–
Ethnicity					
Asian	2 (0.4%)	0 (0%)	2 (0.8%)	–	–
Black	7 (1.4%)	3 (1.1%)	4 (1.7%)	–	–
Mixed	4 (0.8%)	2 (0.7%)	2 (0.8%)	–	–
Unknown	8 (1.6%)	3 (1.1%)	5 (2.1%)	–	–
White	488 (95.9%)	261 (97.0%)	227 (94.6%)	–	–
Age at diagnosis					
Mean (SD)	63.7 (9.53)	63.7 (9.53)	–	–	–
Median [Min, Max]	65.5 (28.3, 80.5)	65.5 (28.3, 80.5)	–	–	–
Age at assessment					
Mean (SD)	61.4 (6.40)	61.5 (6.61)	61.4 (6.16)	–	
Median [Min, Max]	63.0 (40.0,70.0)	64.0 (40.0,70.0)	62.5 (40.0,70.0)	–	–
Age (median [IQR])				67 (60–71)	67 (62–75)

Regarding our validation dataset, *Bosch* et al. had 33 participants (13 cancer patients and 20 controls) with a median age of 67 among colorectal cancer participants which included 6 male participants (**Table 2**). For the downstream analysis, 518 samples and 2923 proteins were assessed for UK Biobank, and 33 samples with 521 proteins made up the validation dataset *Bosch et.al*.

### 3.2 Univariate analysis

**Figure 2:**
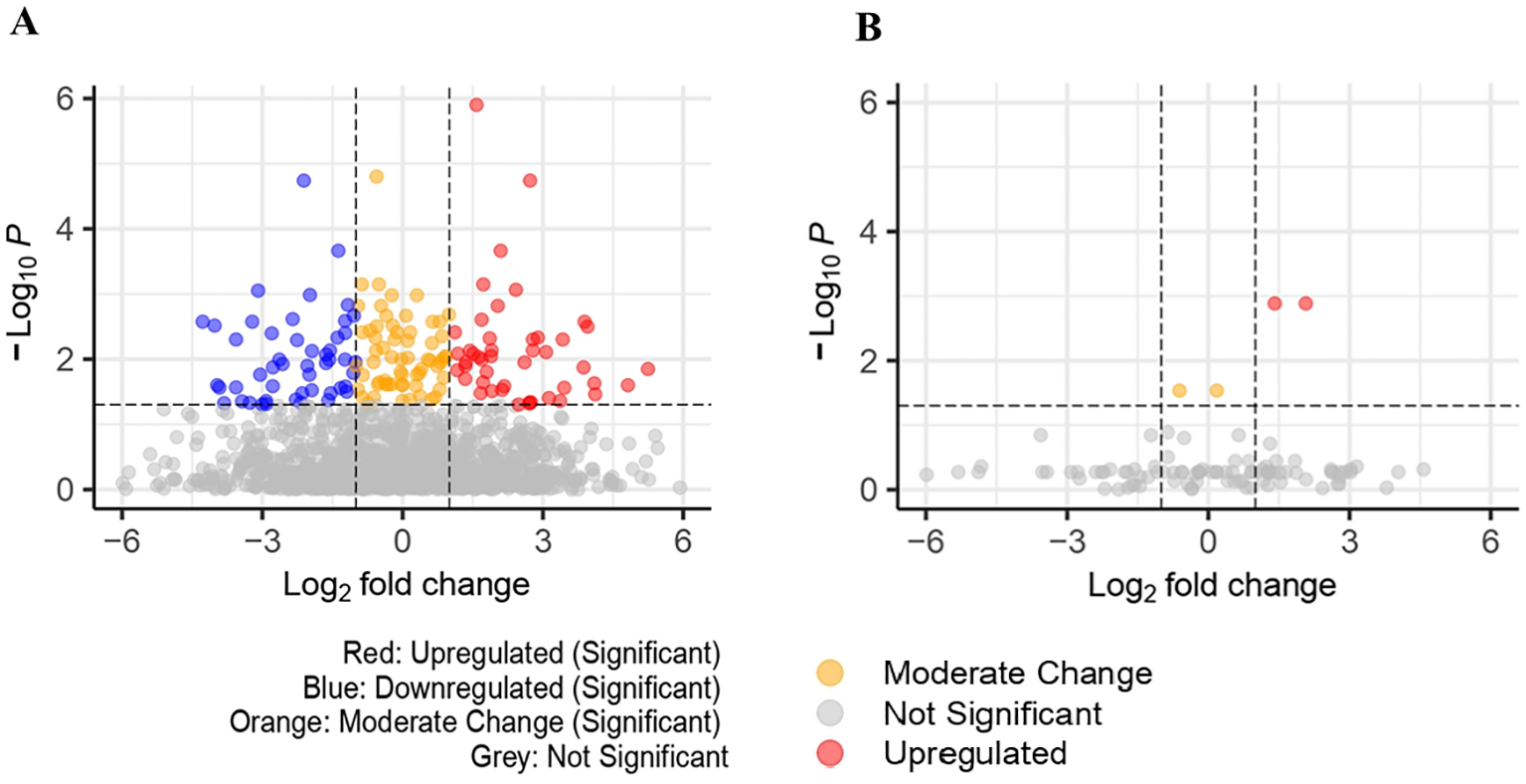
**(A)** Volcano plot for significant proteins(padj<0.05) in UK Biobank dataset (159 significant proteins). **(B)** Volcano plot for significant proteins (padj<0.05) in *Bosch* et al. dataset (4 significant proteins).

Univariate comparisons between cases and controls found 159 significant proteins (FDR<0.05) in UK Biobank and 4 significant proteins in *Bosch et al.*, however, we were unable to identify any significant proteins in common among the datasets. These are represented in **Figures 2A, B** volcano plots, showing the upregulated and downregulated proteins among the significant proteins. Proteins found in the univariate analysis were used in the phases of the following analysis, with the 159 from UK Biobank used in the first phase and the 98 of those that were also found in *Bosch* et al. used in the second phase, since these would be used in the validation tests.

### 3.3 Performance evaluation

The performance of the classifiers was assessed in both phases using eXtreme gradient boosting (XGBoost), light gradient boosting machine (LGBM), and least absolute shrinkage and selection operator (LASSO). **Supplementary Tables S2** and **S3** summarize the hypertuning parameters used. **Supplementary Tables S4** and **S5** summarize the performance metrics for each classifier.

With the objective to boosting model accuracy, reducing overfitting, and improving generalization on unseen data, each model’s hyperparameters were defined in specific grids to allow GridSearchCV to test a range of potential values. For XGBoost, the parameters included n_estimators to control boosting rounds, max_depth to set tree complexity, learning_rate to balance learning speed and accuracy, and subsample and colsample_bytree to reduce overfitting through data and feature sampling. Similar parameters were used by LightGBM, but the complexity of each tree was controlled by num_leaves rather than n_estimators. The regularization strength affects feature selection by reducing coefficients towards zero, hence we only defined the alpha parameter for Lasso regression. So as to maximize AUC (Area Under the ROC Curve), a metric that assesses the trade-off between true positive rate (TPR) and false positive rate (FPR) and is especially well-suited to binary classification, we analyzed each model’s performance across these grids and identified the best parameter combinations. GridSearchCV was set up with stratified 5-fold cross-validation using StratifiedKFold for every model. In order to ensure stability and prevent overfitting to any specific data split, this approach splits the data into five parts while preserving class proportions in each fold. GridSearchCV evaluates every parameter combination over all folds using AUC as the evaluation measure. It then chooses the combination that has the greatest average AUC score, hence determining the optimal model configuration. By avoiding overfitting and offering an unbiased evaluation of the model’s performance on the training set, this cross-validation technique enhances the generalizability of the chosen parameters. After determining the optimal parameters, we assessed each model’s performance in the test set. AUC, specificity, sensitivity, precision, and F1-score are among the metrics we calculated to give a thorough evaluation of each model’s strengths and weaknesses. The confusion metrics for each dataset of the two phases in shown in **Supplementary Tables S6** and **S7**.

The classifiers produced quite similar performance metrics for the UK Biobank dataset. LASSO generated the highest AUC test score of 0.75 (95% CI:0.65-0.84), whereas XGBoost scored 0.71 (95% CI:0.61-0.81) and LightGBM 0.70 (95% CI:0.59-0.79). With an optimal grid alpha value for LASSO of 0.01, it was the most effective at detecting individuals with colorectal cancer. LASSO further exhibited some balance between recall and precision with an F1 score of 0.73. The model’s specificity score of 0.67 suggests that it has the potential to accurately detect non-CRC cases. However, the model exhibited more errors in accurately comprehending non-CRC cases (**Supplementary Figure S4A**). In the second phase, LASSO produced an AUC test score of 0.64 (95% CI:0.52-0.74), again the best of the three classifiers, while XGBoost scored an AUC of 0.63 (95% CI:0.52-0.73) and LightGBM with AUC of 0.60 (95% CI:0.49-0.70). Lasso had an optimal grid value of 0.01 and an F1 score of 0.61. The specificity score of the model of 0.42 indicates that it had little ability to accurately identify instances that were not colorectal cancer (**Supplementary Figure S4B**).

Performance of the classifiers was markedly worse in the validation dataset (*Bosch et.al*). When the classifier’s performance was compared, XGBoost and LightGBM performed better with an AUC test of 0.53 (95% CI:0.41-0.66) than LASSO (AUC test = 0.40(95% CI:0.20-0.63). XGBoost and LightGBM detected all positive instances (recall=1) but failed to find any negative predictions (specificity=0) (**Supplementary Figure S5A**). In phase 2, XGBoost was found to be performing moderately with an AUC test score of 0.61 (95% CI:0.40-0.82) compared to LightGBM AUC test 0.57 (95% CI:0.36-0.77) and LASSO AUC test 0.55 (95% CI:0.35-0.74). The optimal grid parameters of XGBoost were colsample_bytree:0.8, learningrate:0.05, max_depth:5, n_estimators:200, and subsample:0.8. It produced an F1 score of 0.59, which reflects a balance between recall and precision, demonstrating XGBoost was successful in differentiating between colorectal cancer and non-colorectal cancer (**Supplementary Figure S5B**).

### 3.4 Feature selection and interpretation

**Figure 3:**
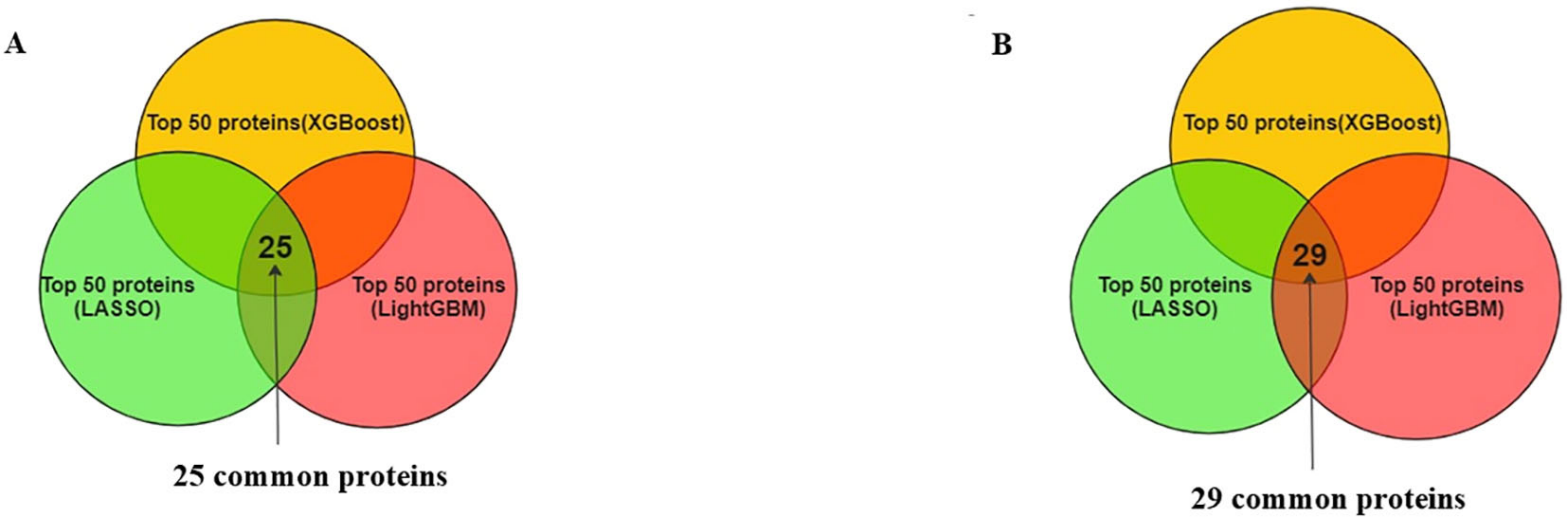
**(A)** Common protein (25) among top 50 proteins (3 models) of UK Biobank. **(B)** Common proteins (29) among top 50 proteins (3 models) of UK Biobank.

**Figure 4:**
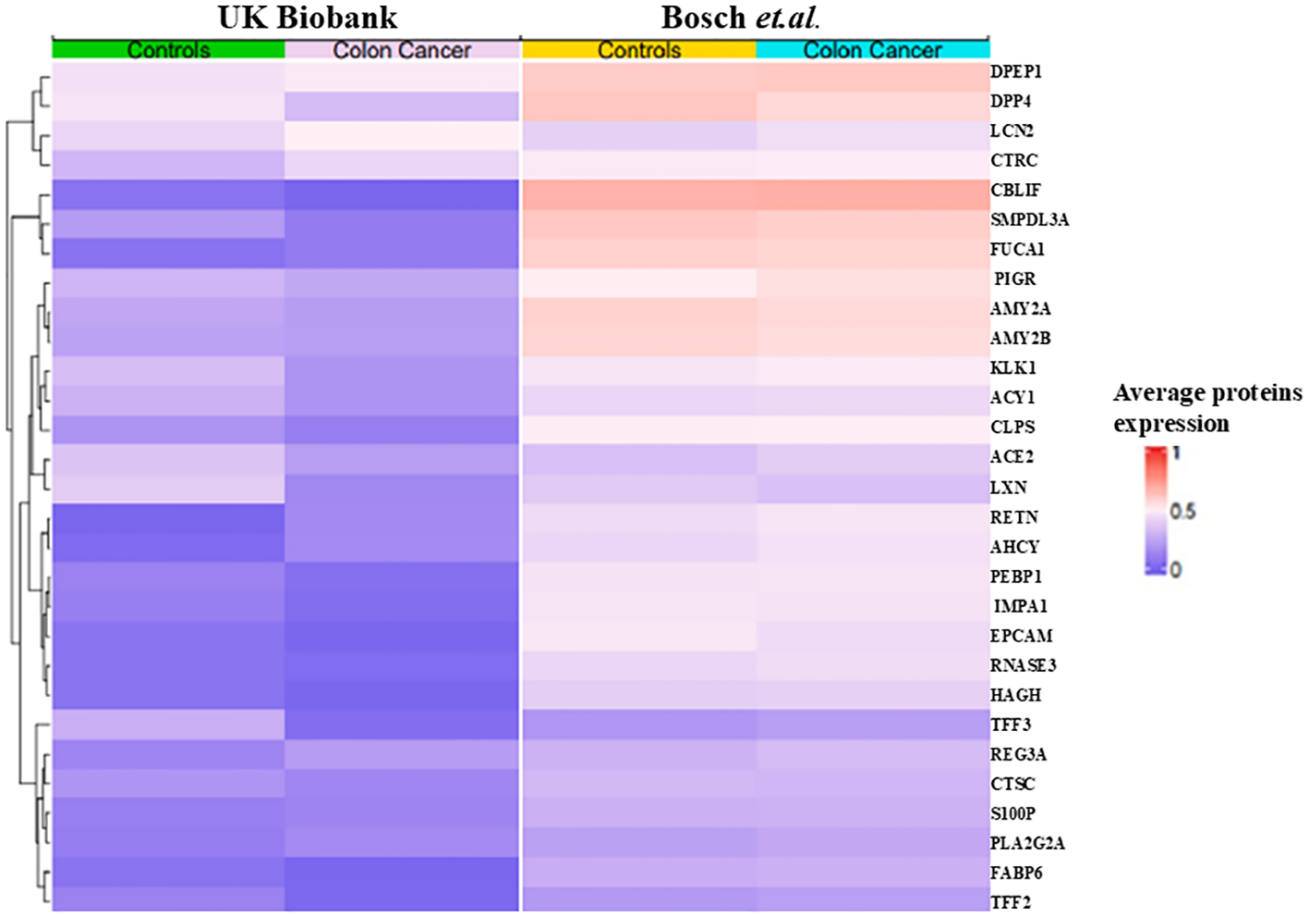
Heat map of 29 proteins of UK Biobank and *Bosch et al*.

Analysis using SHAP identified 25 proteins (**Figure 3A**) that were shared among the top 50 proteins in three models in the first phase of UK Biobank data. Of these, two proteins were shared with those found in *Bosch* et al. After extracting the 98 proteins of UK Biobank in phase 2, 29 of the top 50 proteins of each model were common among three classifiers (**Figure 3B**). These same 29 proteins were extracted from *Bosch* et al. and validated using the three models. The 29 common proteins of both datasets were visualized using a heatmap, as seen in **Figure 4**. One notable difference is that CBLIF and DPEP1 were expressed less in cases found in UK Biobank but higher in *Bosch* et al. cases.

In the first phase, the most important protein was discovered to be the inflammation indicator (63) CEACAM5 in both datasets followed by B4GAT1, MFAP3, and LRRN1 were the next most highly ranked proteins in the UK Biobank data whereas MZB1, and ACE2 were determined to be low ranked proteins. From examining the local model impact plots, CEACAM5, B4GAT1, MFAP3, and LRN1 were observed to influence the model’s prediction of the predictive class CRC. On the other hand, MZB1and ACE2 appeared to be predictive of a lower likelihood in the prediction of colorectal cancer. PLA2G2A did not show up much expression in the *Bosch* et al. data (**Supplementary Figures S6A–D**).

**Figure 5:**
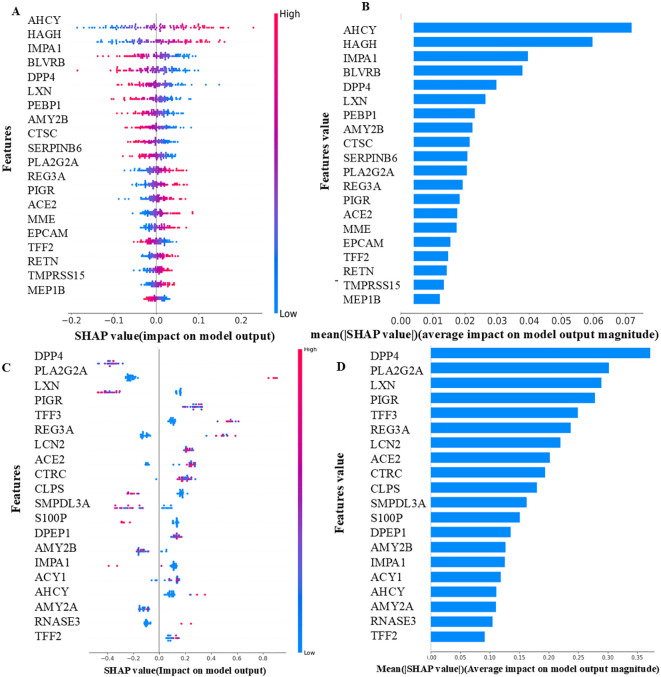
**(A)** UK Biobank - Local importance (LASSO-Phase 2). **(B)** UK Biobank - Global importance (LASSO-Phase 2). **(C)** *Bosch* et al. - Local importance (XGBoost-Phase 2). **(D)** *Bosch* et al. *-*Global importance (XGBoost-Phase 2). The features displayed are the top 20 proteins of each model, as determined by their SHAP values. The samples are shown as colored dots in the local importance summary plot for SHAP values, the color of each dot corresponds to its value for that feature. Positive SHAP values have a positive effect on the model and direct the algorithm to predict the positive class, and vice versa. In the global importance summary plot for mean absolute SHAP values higher rank features are associated with more samples having SHAP values.

The most important proteins found in UK Biobank in phase 2 were discovered to be AHCY and HAGH. Also notable are TMPRSS15 and MEP1B considered less important in *Bosch* et al. There was higher expression of DPP4 and PLA2G2A in individuals with CRC and the least important protein was TFF2. The proteins AHCY, HAGH, DPP4, and PLA2G2A had elevated levels and were chiefly responsible for the prediction of CRC. TMPRSS15, MEP1B, and TFF2 lowered the prediction of CRC (**Figures 5A–D**).

### 3.5 Interaction networks using STRING and the Human Protein Atlas

The proteins common in the two datasets were given as input into the STRING database to identify interactions. The protein CD163, one of the 25 proteins (**Supplementary Figure S7A**) identified in the first phase, was shown to have a strong correlation with other proteins, including IL2RA, HGF, LAG3, SELE, and to be crucial for both cell adhesion and metastasis. The inflammatory protein CEACAM5 had a strong correlation with TFF1 and LAG3 proteins. Analysis of 29 proteins (**Supplementary Figure S7B**) in phase 2 revealed that AMY2A and AMY2B had evidence of a strong interaction in pancreatic cancer, while our study did not find a significant correlation between them. Experimentally, it was discovered that TFF3 and TFF2 were highly correlated.

Seven proteins, TFF3, LCN2, CEACAM5, TFF1, SELE, RETN, and AHCY were found to be upregulated in other databases and also in our UK Biobank where TFF3 and LCN2 were found to be the most significant proteins and were highly expressed in colorectal cancer. The comparison of seven proteins in the case and control groups is illustrated (**Supplementary Figures S8A–G**).

The prediction of both dataset models was tested with SHAP XAI, which also highlighted the key proteins influencing the prediction of the models. The top proteins found in the UK Biobank included CEACAM5, B4GAT1, and AHCY. However, the model’s average AUC score of 0.69 implies that these proteins are not effective in classifying colorectal cancer samples in UK Biobank and cannot distinguish between CRC cases and controls. However, given that the results are not as low as chance 0.50, it is acceptable to make the assumption that the pathophysiology of CRC is influenced by these important proteins, with findings supported by the literature mentioned below. According to previous studies CEACAM5 (64), B4GAT1 (65), and AHCY (66) were discovered to be biomarkers of CRC due to their strong involvement in the methylation and inflammatory processes of tumor cells. These were discovered to be inverse biomarkers that correspond with the CRC prediction made by our UK Biobank models.

Seven proteins namely TFF3, LCN2, CEACAM5, TFF1, SELE, RETN, and AHCY proteins of both phases were found to be upregulated in colorectal cancer in human protein atlas database, also were significant in our UK Biobank dataset and had an essential function in CRC (**Supplementary Table S8**).

TFF3 and LCN2 were found to be the top most significant proteins in the UK Biobank.TFF3 and LCN2 were found to be highly expressed in other cancers like Myeloma, and lung cancer in the human protein atlas database.TFF3 was the most significant protein found in our study and played an important role in the proliferation, migration, and invasiveness of HT29 cells in colorectal cancer (67).TFF3 has a physical association with PCBD1, UBQLN2, and SGTA which were considered to be transcription factors and other protein classes in the human protein atlas database (**Supplementary Figure S9A**). TFF3 plays an important role in the apoptosis, and cell proliferation along with the promotion of angiogenesis in colorectal cancer (68) (**Supplementary Figure S9B**). It interacts with tyrosine kinase (src) protein and activates signal transducer and activator of transcription 3 (STAT3) which is plays an important role in the signaling pathway of cancer progression (69). A genome-wide association study (GWAS) analysis revealed that chromosome 21 is known to have the TFF3 protein. The expression of TFF3 was found to be high in our CRC samples.

LCN2 appeared to be the 2^nd^ most significant protein and played an important role in the enzymatic activity of matrix metalloprotease-9 causing metastasis of colorectal cancer cells (70) It has a physical association with several other transcription factors and other protein classes (**Supplementary Figure S10A**). They played an important role in tumor cell growth, iron toxicity, and methylation and served against the anti-cancer drugs (71) (**Supplementary Figure S10B**). GWAS analysis revealed that chromosome 9 is known to have the LCN2 protein.

### 3.6 Weighted gene co-expression network analysis results

In our assessment of the UK Biobank dataset, we evaluated the scale-free topology model fit and mean connectivity to determine its suitability for WGCNA. Although the mean connectivity was not atypical, the consistently low signed R² values indicated a poor fit to the scale-free topology model (**Supplementary Figures S12A, B**). This suggested that the dataset did not fully exhibit the scale-free network characteristics typically required for robust co-expression analysis. As a result, the dataset was deemed unsuitable for further WGCNA analysis and no modules were identified or additional analyses, such as module-trait correlations or functional enrichment were performed.

**Figure 6:**
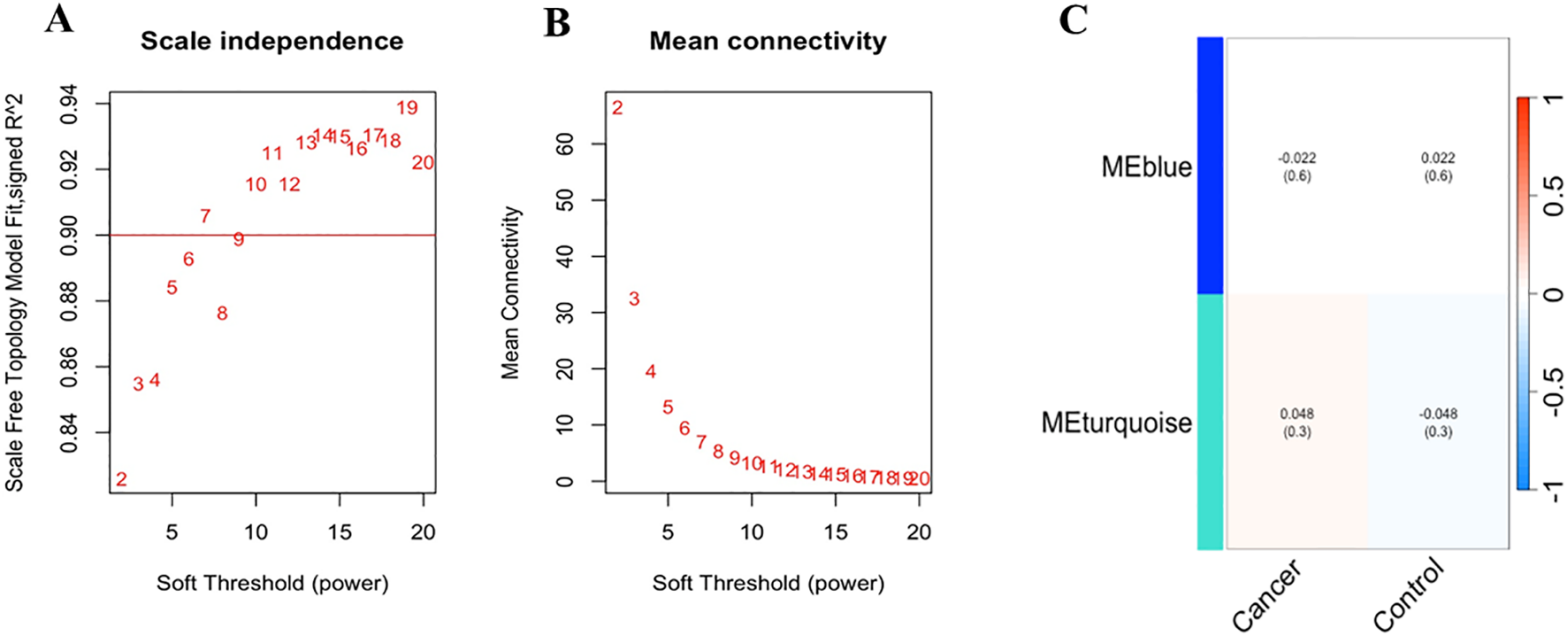
**(A)** Scale-free topology model fit and mean connectivity analysis of *Bosch et.al*., dataset: The horizontal line at a signed R² value of 0.90 indicates that the network exhibits a good scale-free topology fit across the tested range of soft-thresholding power (β) values, suggesting that the network’s degree distribution closely follows a power-law distribution. **(B)** The mean connectivity for different β values shows as β increases, the mean connectivity decreases, indicating a reduction in the number of connections per node, which is consistent with a sparser network structure. The selected optimal β value of 8 balances. **(C)** The heatmap illustrates the correlation between the identified module eigengenes (MEs) and the defined traits, “Cancer” and “Control” (non-cancer). The rows represent the MEs (MEblue and MEturquoise), and the columns represent the traits. Each cell contains the Pearson correlation coefficient, with the corresponding p-value provided in parentheses.

In the *Bosch* et al. dataset the chosen β value of 8 was supported by a scale-free topology model fit (signed R²) consistently achieving a value of 0.90 across tested β values, indicating a robust scale-free network (**Figure 6A**). The mean connectivity plot showed a decline with increasing β, suggesting a sparser network structure, with the selected β ensuring a balance between scale-free topology and network interpretability (**Figure 6B**).

Subsequently, module detection via hierarchical clustering on the *Bosch* et al. dataset identified two distinct modules, MEBlue and METurquoise, with the remaining genes assigned to the Gray module, indicating no significant co-expression. The cluster dendrogram visually confirmed these modules, with MEBlue and METurquoise displayed prominently (**Supplementary Figure S11**).

To explore the functional relevance of the identified modules, we correlated the module eigengenes (MEs) with the defined clinical traits (**Figure 6C**). The MEBlue module exhibited a low negative correlation with cancer (r = -0.022, p = 0.6) and a low positive correlation with control (r = 0.022, p = 0.6), suggesting no significant differential expression in relation to these traits. In contrast, the METurquoise module showed a slightly positive correlation with cancer (0.048, p = 0.3) and a slightly negative correlation with control (r = -0.048, p = 0.3), indicating a marginal trend of up-regulation in cancer samples.

Further functional annotation through KEGG pathway enrichment analysis revealed that genes in the METurquoise module were significantly associated with several biological pathways, with the MAPK signaling pathway being the most enriched. This suggests a potential involvement of these genes in critical cellular processes such as proliferation and survival, which is relevant to cancer pathology.

Overall, our WGCNA analysis has identified key gene modules and potential functional pathways associated with cancer. While the correlations with clinical traits were modest, the identification of the signaling pathways highlighting potential biological mechanisms calls for further investigation. This experiment provides a comprehensive framework for understanding gene co-expression patterns and their potential functional implications in colorectal cancer.

### 3.7 Pathway-based interpretation

**Figure 7:**
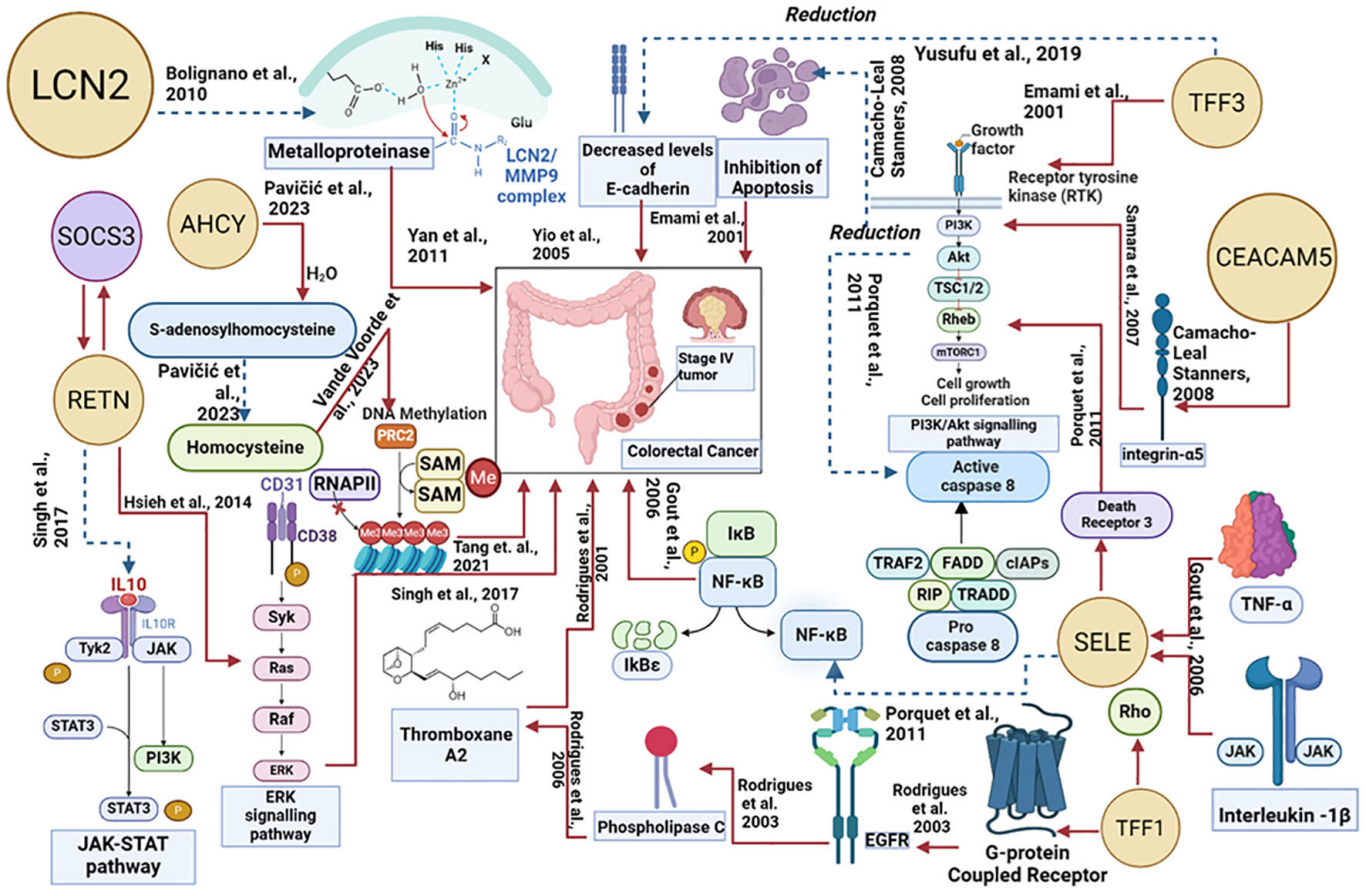
Proteogenomic insights into the biological pathways of proteins associated with colorectal cancer. The red line denotes the upregulated actions, and the blue dotted lines denote the downregulated actions.

**Figure 7** explains how each of the biomolecules is capable of triggering colorectal cancer through various proteogenomic pathways. Trefoil factor 3 (TFF3) has been found to promote cell migration and increase proliferation of colorectal cancer cells. Overexpression of TFF3 in colorectal cancer cells decreases the levels of E-cadherin which results in increased epithelial-mesenchymal transition, enhancing colon cell migration and promoting the formation of secondary tumors, thereby progressing cancer (67). Decreased levels of E-cadherin activate the EGF receptor signaling cascade leading to phosphorylation of β-catenin and activation, altering cell-cell interactions and leading to cell migration (72). TFF3 has also been found to activate signaling pathways that promote cellular invasion including src/RhoA, PI3K/Akt, and phospholipase C (PLC)/Protein kinase C (PKC) pathways. By activating the PI3K/Akt signaling pathway, cell survival and invasion are enhanced (73). Activation of this pathway also leads to downstream effects such as inhibition of pro-apoptotic factors and activation of proteins that promote protein synthesis and cell growth. The PLC/PKC pathway is also involved in cellular motility and invasion, which is an important step in the metastatic spread of cancer cells (73). Compared to healthy cells, in colorectal cancer cells levels of Lipocalin 2 (LCN2) are elevated (74). In colorectal cancer cell lines LCN2 overexpression was linked to increased invasion of cells and loss of cell-to-cell adhesion (74). LCN2 has been shown to protect matrix metalloproteinase 9 (MMP9) from proteolytic degradation by forming an LCN2/MMP9 complex (74). MMP9 plays an important role in the resorption of the extracellular matrix and therefore in metastasis and neoplastic invasion (75). By covalently bonding to MMP9, LCN2 can decrease the degradation of MMP9 and therefore increase tumor progression by enhancing the enzymatic activity of MMP9 (75, 76). Carcinoembryonic antigen-related cell adhesion molecule 5 (CEACAM-5) is a glycoprotein overexpressed in colorectal cancer (77). CEACAM-5 inhibits anoikis, a type of apoptosis that is triggered by the loss of extracellular matrix-cell contacts, therefore disrupting colonic tissue architecture (77, 78). CEACAM5 interacts with DR5, a member of the death receptor family found on the plasma membrane of colorectal cancer cells that have detached from the extracellular matrix. This leads to reduced caspase-8 activation therefore leading to the inhibition of caspase-3 (79). CEACAM-5 is clustered in lipid rafts and activates integrin-α5 which activates the PI3k/Akt signaling pathway which promotes cell survival. Downstream effects of activating the PI3K/Akt signaling pathway include inhibition of the release of cytochrome-c from the mitochondria, resulting in the prevention of apoptosis in detached cells and increasing migration of the cell allowing the formation of a secondary tumor (78).

Trefoil factor 1 (TFF1) overexpression facilitates tumor growth and invasiveness through various pathways including Rho-GTPases, Rho-kinase, PI3-kinase, PLC, and COX-2 (73, 80).TFF1-induced cellular invasion is dependent on the EGFR signaling pathway.TFF1 indirectly activates EGFR, through a mechanism involving the transactivation of EGFR using G-protein coupled receptors (GPCRs) (73)TFF1 can also increase invasiveness through Thromboxane A2 (TXA2) receptor/PLC - dependent mechanisms. TFF1 upregulates the production of TXA2 which binds to the TXA2 receptor which is coupled to G-proteins and goes on to activate PKC. PKC enhances cell proliferation, survival, and invasion therefore progressing colorectal cancer (73). Selectin-E (SELE) levels are significantly higher in colorectal cancer cells in comparison to adjacent healthy cells (55). SELE is induced by pro-inflammatory stimuli such as tumor necrosis factor-α and IL-1β (81, 82). SELE is able to bind death receptor 3 (DR3) and activate it leading to the recruitment of src kinase. Src kinase phosphorylates the tyrosine residues on DR3 leading to activation of PI3K. PI3K activates Akt leading to a variety of downstream effects including the activation of the NFκB pathway. The PI3K/Akt/NFκB pathway is known to protect colorectal cancer cells from apoptosis by reducing the activity of caspase-8 and caspase-3. These caspases are important in the induction of apoptosis, suggesting that SELE increases the survival of cells and resistance towards apoptosis, further progressing the tumor (82). Resistin (RETN) is overexpressed in colorectal cancer (83). RETN binds to toll-like receptor 4 (TLR4) on the surface of colorectal cancer cells, switching on ERK signaling (83, 84). Activated ERK can promote upregulation of the gene SOCS3 which leads to decreased phosphorylation of JAK2 and STAT3 (83). Inhibition of the JAK2/STAT3 signaling pathway results in the growth arrest of cells in the G1 phase of the cell cycle, therefore regulating cell growth (83). S-adenosylhomocysteine hydrolase (AHCY) levels in colorectal cancer are disrupted, leading to an imbalance in methylation processes. AHCY is an enzyme that catalyzes the hydrolysis of S-adenosylhomocysteine (SAH) into homocysteine. Increased activity of AHCY leads to increased conversion of SAH into homocysteine. SAH inhibits the activity of methyltransferases and as AHCY inhibits the accumulation of SAH, there is more DNA methylation occurring leading to abnormal gene expression which contributes to tumor progression (66).

### 3.8 Protein quantitative trait loci analyses results

Significantly associated loci were identified in the groups with prior CRC and that would go on to be diagnosed with CRC after sampling. In those who had been previously diagnosed with CRC, this included 57 SNPs associations with SELE levels and for those who went on to be diagnosed, there were 67 SNPs associated with CEACAM5, 136 with SELE, and 1 with TFF1. However, all of these points were more significantly associated by p-value in the control group and in SNPs whereby significant association was found in a CRC cohort, the Beta coefficient always had the same sign as in the control cohort, indicating the SNP significantly associated had a similar effect to the control group (**Supplementary Figure S13**, **Supplementary Table S9**).

### 3.9 Impact of batch-effect correction on model outcomes

A harmonized dataset of 98 common proteins from the UK Biobank and *Bosch* et al. databases were used to evaluate the performance of prediction models such as XGBoost, LightGBM, and LASSO regression. AUC was 0.60, specificity was 0.58, recall was 0.57, accuracy was 0.59, and F1 was 0.58 for LASSO regression. The AUC score for both XGBoost and LightGBM was 0.57. XGBoost’s specificity was 0.52, sensitivity was 0.62, precision was 0.58, and F1 score was 0.60. LightGBM’s specificity was 0.49, recall was 0.66, precision was 0.57, and F1 score was 0.61.

LightGBM obtained an AUC score of 0.62, specificity of 0.52, recall of 0.66, accuracy of 0.59, and F1 score of 0.62 for the seven potential biomarkers. An AUC score of 0.60, specificity of 0.49, recall of 0.62, accuracy of 0.56, and F1 score of 0.59 were all attained with LASSO regression. With an F1 score of 0.59, recall of 0.60, accuracy of 0.57, specificity of 0.52, and AUC of 0.57, XGBoost performed well. The proteins AHCY, LCN2, CEACAM5, TFF3, TFF1, SELE, and RETN were shown to be the most significant when the highest AUC model LightGBM model was subjected to SHAP analysis to ascertain feature importance, suggesting their possible applicability in predictive modeling. The distribution of the seven proteins of the ComBat-corrected dataset is depicted (**Supplementary Figure S14**).

#### 3.9.1 Comparative validation of potential biomarkers across CRC studies

The expression levels of TFF3 and TFF1 in colorectal cancer tissues were examined in a study conducted by Yusufu et al. (67) which revealed TFF3 and TFF1 expression levels are elevated in colorectal cancer and promote the malignant behavior of colon cancer by activating the EMT process. The mRNA expression levels of these proteins were assessed by the researchers using quantitative real-time PCR (qRT-PCR). They found that TFF3 and TFF1 expression was elevated with a fold change of 3.5 and 2.8, respectively when compared to nearby normal tissues. These results imply that increased TFF3 and TFF1 levels could be a factor in colon cancer’s aggressive behavior, Li et al. (85) **analyzed** the expression of the SELE gene using tissue samples from normal tissues and colorectal cancer (CRC) tissues. Quantitative reverse transcription polymerase chain reaction (qRT-PCR) was the primary technique used to assess the levels of expression. The results showed that, with a reported fold change of roughly 2.5, SELE expression was considerably elevated in CRC tissues relative to normal tissues. SELE may be a useful biomarker for colorectal cancer prognosis, providing information about tumor biology and possible treatment targets, according to these findings. The Vande Voorde et al. (66) study on AHCY utilized tissue samples from mutated mouse models of colorectal cancer (CRC). The researchers used multimodal mass spectrometry-based technology. They found that gene adenomatous polyposis coli-deficient CRC tissues had 3.2 times higher levels of S-adenosylhomocysteine and 2.5 times higher levels of S-adenosylmethionine than normal tissues. To examine the role of lipocalin2 (LCN2) in colorectal cancer (CRC), the study by Feng et al. (86) used a variety of cutting-edge technologies. Real-time PCR, Western blotting, and immunohistochemistry (IHC) on 400 CRC tissue samples were among the methods employed to evaluate the amounts of mRNA and protein. The functional roles of LCN2 in carcinogenesis and metastasis were investigated using mice xenograft models in conjunction with other assays such as colony formation, immunofluorescence, wound healing, migration, invasion, and luciferase reporter assays. The researchers discovered a substantial differential in LCN2 expression, with a fold change above 20 (P < 0.01). The study by Gisina et al. (87) on CEACAM5 overexpression, used 30 colorectal tumors in an effort to find biomarkers of CD133-positive cases. Using label-free quantification for comparative proteome profiling, researchers used liquid chromatography-tandem mass spectrometry (LC-MS/MS) to identify proteins that were differentially expressed. CEACAM5 and other identified proteins were confirmed to exhibit differential expression using flow cytometry and the enzyme-linked immunosorbent assay (ELISA). Results indicated that CEACAM5 was consistently overexpressed in CD133-positive cells across all samples, with fold differences average of 18 (ranging from 3.4 to 32.6). This suggests that CEACAM5 is a reliable marker for colorectal patients that are CD133-positive. The role of Resistin in colorectal cancer has been researched in a study by Rompou et al. (88), which compared the expression levels of Resistin (RETN) in CRC tissues with those in normal tissues. They used the enzyme-linked immunosorbent assay (ELISA) to evaluate the protein concentrations and the quantitative real-time polymerase chain reaction (qRT-PCR) to determine the levels of Resistin mRNA. The results demonstrated that Resistin expression was much higher, with a fold shift of about 2.5, and statistically significant (p < 0.01) when compared to normal tissues in CRC samples. The fold change value of each study is depicted (**Supplementary Figure S15**).

## 4 Discussion

### 4.1 Model performance

In this study, we compared three algorithm’s ability to distinguish between individuals with CRC and controls, based on proteomics data from two sources. However, LASSO surpassed the other machine learning models used in the UK Biobank. This is probably because the LASSO model, which automatically removes strongly correlated variables, gave zero weights to a number of potentially redundant features that were included in the models. When integrating the results from the two phases, LASSO consistently showed better performance in predicting colorectal cancer compared to XGBoost and LightGBM with some variability in specificity. With test AUC of 75.05% in phase 1 and 63.37% in phase 2, LASSO achieves the highest test results overall, suggesting a superior capacity to differentiate between cases of colorectal cancer and non-colorectal cancer cases. The test AUC for XGBoost and LightGBM, which are 71.42% and 69.61% in phase 1 and 62.25% and 60.35% in phase 2 respectively are significantly lower than LASSO. LASSO dominates with an F1 score of 73.87% in Phase 1 and 61.01% in Phase 2, which balances recall and precision. This suggests that LASSO, as compared to XGBoost and LightGBM, is more successful in accurately predicting cases of CRC while minimizing false positives and false negatives. LASSO achieves 71.92% in Phase 1 and 56.25% in Phase 2, exhibiting high precision. Although precision reduces in Phase 2, LASSO outperforms Light GBM and XGBoost with recall values of 75.92% in Phase 1 and 66.66% in Phase 2. LASSO had a strong ability to predict true positive classes, showing its superior capacity to identify a larger proportion of actual cases of CRC. Variability is shown in specificity, which reflects the ability of the model to identify real negatives. In Phase 1, LASSO achieves 67.34%, but in Phase 2, it falls to 42.85%. LASSO was surpassed in Phase 2 by XGBoost and LightGBM, with specificity of 52.01% and 53.06, respectively. This suggests that although LASSO is very good at detecting actual positive instances, it could generate more false positives than LightGBM and XGBoost, particularly in phase 2.

On the other hand, models did not play well in the *Bosch* et al. data. Comparatively, XGBoost performed better but with a moderate accuracy rate in predicting CRC cases with some variability in specificity. Phase one results show that LASSO falls behind at 40%, while XGBoost and LightGBM have the greatest test AUC of 53%. LASSO comes in at 55.38%, LightGBM at 57.30%, and XGBoost at 60.76%, all of which continue to lead in Phase 2. By 57% in Phase 1 and 59.99% in Phase 2, XGBoost also leads in the F1 score. The findings indicate that XGboost outperforms LASSO and LightGBM in accurately predicting CRC while minimizing false negatives and positives. LightGBM and XGBoost have the same F1 score in phase 1, however, in phase 2, LightGBM’s score drastically decreases to 34.78%. Phase 1, LASSO F1 score is 57%, however, phase 2 score is far lower at 23.5%. In Phase 2, XGBoost has the highest precision of 52.94% among the predicted positives, whereas both LASSO and LightGBM have the lowest precision. LightGBM precision is 40% while LASSO increases to 50% in phase 2. All models in Phase 1 exhibit 100% recall, a measure of their ability to detect true positive CRC patients. In Phase 2, LightGBM comes in second at 30.76%, LASSO at 15.38%, and XGBoost is leading once more at 69.23%. With LASSO in phase 2, the ability of the model to identify the true negative class is highest with specificity of 90%, followed by LightGBM at 70% and XGBoost at 60%. Phase 1 results demonstrate that all models have 0% specificity, meaning that none of them can accurately identify true negative classes.

These results emphasize the significance of choosing a prediction model that balances the trade-offs between precisely identifying true positive and negative cases while retaining high accuracy.

Finally, we compared the similarities and differences between our study and the previous studies. A prior study (89) used mass spectrometry profiling combined with machine learning techniques to develop an understanding of the molecular mechanisms underlying the progression of colorectal adenomas into colorectal cancer. With elastic net regression and a set confidence range of 0.7–0.95, it remarkably revealed a greater model accuracy of 90% while maintaining a balance between specificity and sensitivity. The higher performance could be related to many factors, such as the comprehensive nature of the protein data examined and the use of modern profiling technologies. The type and amount of data that were available may have restricted our study, where we used three separate models. Although our models had some degree of effectiveness, they lacked the specific insights provided by proteomics data. Despite the modest sample sizes in both investigations, their research highlights the benefits of sophisticated model approaches and advances in proteome profiling.

This study utilized the ComBat batch-effect correction method to successfully harmonize protein expression data from separate cohorts: *Bosch* et al. and the UK Biobank. This approach preserved significant biological variability while successfully reducing batch effects brought on by variations in cohort characteristics and data collection methods. By ensuring that the model’s performance was not influenced by the data’s source, the harmonized dataset provided a strong basis for subsequent predictive assessments. Nevertheless, there was no discernible difference in the models’ overall predictability when compared to analyses carried out without batch-effect correction, indicating that batch effects might have had little effect on this particular dataset. LASSO regression, LightGBM, and XGBoost are three machine learning models that were employed to predict results based on data on protein expression. These models’ AUC ratings, which ranged from 0.57 to 0.62, showed moderate prediction performance across the harmonized dataset. While LASSO regression may not be able to fully capture non-linear data correlations, it demonstrated steady and balanced performance, obtaining an AUC of 0.60 for the whole dataset and for selection of seven important biomarkers. The AUC values of the gradient-boosting techniques LightGBM and XGBoost were similar, but LightGBM outperformed XGBoost in terms of sensitivity and F1 scores, especially when used on the biomarker sample. The potential use of seven proteins as biomarkers was emphasized by their identification as significant predictors: AHCY, LCN2, CEACAM5, TFF3, TFF1, SELE, and RETN. Their relative relevance was highlighted by SHAP analysis, which also shed light on how they affect model predictions. By concentrating on these biomarkers, LightGBM in particular showed enhanced performance (AUC: 0.62, F1: 0.62), highlighting its capacity to give priority to significant features in intricate datasets. Even though none of the models produced very good prediction results, the results indicate that batch-effect correction is still essential for guaranteeing the accuracy and interpretability of results even though it may not always result in appreciable gains in model performance. These findings highlight the identified proteins’ potential as biomarkers and call for more research to fully understand their biological importance and predictive modeling capability.

### 4.2 Proteins identification and clinical aspects

The predictions of both cohort models were well explained by SHAP XAI, which also revealed the important features of both datasets. CEACAM5 and AHCY were UK Biobank’s top proteins. However, the average AUC score of 69.21 suggests that these variables are not highly successful in classifying CRC patients within the UK Biobank. The results are not as low as chance 0.50, thus it is possible to assume that these top proteins are involved in the pathophysiology of colorectal cancer. This finding is supported by literature. An earlier study, for example, revealed that CEACAM5 is implicated in hypomethylation, which results in enhanced expression of carcinoembryonic antigen. This alters the importance of progression in CRC and suggests that CEACAM5 is a good biomarker (64). The accumulation of S-Adenosyl-L-homocysteine (SAH) in CRC was also linked to AHCY, which inhibited methylation and encouraged the formation of tumors (66).

TFF3, LCN2, CEACAM5, TFF1, SELE, RETN, and AHCY—the seven important proteins identified in this study were discovered to be relevant and involved in another research. When TFF3 is overexpressed in colorectal cancer cells, transcription factors such Twist1, Snail, and Vimentin are expressed more often while E-cadherin levels are concurrently decreased. Lower levels of E-cadherin lead to an increase in the epithelial-mesenchymal transition which promotes colorectal cancer cell migration and metastases (67). Additionally, it has been discovered that TFF3 activates signaling pathways that support cellular invasion including PI3K/Akt, phospholipase C (PLC)/protein kinase C (PKC), and src/RhoA pathways (73). The architecture of colonic tissue is disturbed by CEACAM-5 by inhibition of anoikis, a kind of apoptosis that is brought on by the loss of extracellular matrix-cell connections (77). Both the intrinsic and extrinsic mechanisms of anoikis can be inhibited by CEACAM-5 which may result in the emergence of a secondary tumor (78).

Overexpression of Trefoil factor 1 is involved in pathways, including Rho-GTPases, Rho-kinase, PI3-kinase, PLC, and COX-2 and they interact with other receptor systems to activate epidermal growth factor receptor EGFR indirectly causing progression of cancer (73). SELE has the ability to bind and activate death receptor 3 (DR3) which in turn activates src kinase PI3K and NFκB pathway. By inhibiting the activities of caspase-8 and caspase-3, the NFκB pathway is known to shield colorectal cancer cells from apoptosis promoting cell survival and resistance to apoptosis (82). Resistin (RETN) binds toll-like receptor 4 (TLR4), activating ERK signaling triggers a variety of downstream signaling events. The p38 MAPK pathway may be activated when RETN attaches to TLR4 on colorectal cancer cells. This causes a variety of genes that are involved in promoting inflammation, cell division, and survival to get phosphorylated which promotes colorectal cancer (83). AHCY prevents SAH from accumulating, and more DNA methylation occurs, which results in aberrant gene expression and aids in the development of tumors (66).

### 4.3 Mechanistic insights into TFF3 and LCN2 in colorectal cancer progression

TFF3 is involved in CRC through its activation of the PI3K/Akt signaling pathway, which is known for promoting cell survival and invasion (90). When activated, PI3K/Akt leads to the inhibition of pro-apoptotic factors, and stimulates mTOR (mammalian target of rapamycin), promoting cell growth and protein synthesis—processes essential for tumor development and metastatic potential in CRC (90). TFF3 binds to CD147, enhancing its interaction with CD44s, which subsequently activates SRC and STAT3 signaling (69). This cascade results in the induction of PTGS2, a gene that fosters cell migration, invasion, and proliferation, all of which are integral to CRC progression (69). LCN2 contributes to CRC progression by forming a complex with MMP9, an enzyme involved in degrading the extracellular matrix (ECM), thus enhancing cancer cell invasion and metastasis (75). As LCN2 inhibits the breakdown of MMP9, its enzymatic activity is enhanced leading to increased invasion and metastasis (75). LCN2 also decreases E-cadherin mediated cell-cell adhesion therefore increasing cell motility and invasiveness through the action of Rac1 in CRC cells (75). LCN2 has also been found to have a role in colitis-mediated colorectal cancer (CAC) (91). Colitis causes there to be an inflammatory environment leading to IL-6 release (91). Il-6 binds to STAT3 upregulating LCN2 in cancer cells (91). NF-κB is also activated in inflamed tissues and also upregulates LCN2 in cancer cells, leading to sustained inflammation (91). This can then lead to PI3K/AKT/mTOR phosphorylation, which helps cancer cells resist apoptosis, increasing cell survival so cancer can progress (91). This multifaceted role of LCN2 in ECM degradation, cell adhesion, and inflammation underscores its significance as both a biomarker and therapeutic target in CRC.

### 4.4 Linking biomarkers to therapeutic strategies

Therapeutically, LCN2 remains challenging to target due to its nonenzymatic nature (70) therefore, current strategies to target LCN2 for therapeutic interventions involve gene editing of LCN2 (70). This is currently observed in the treatment of cholangiocarcinoma (CCA) cells (92). In another study, transfected LCN2-siRNAs were inserted into CCA cells and observed significant decreases in cell invasion and migration but no changes in cell proliferation (92). This suggests a promising translational pathway for future CRC therapies, particularly in patients with advanced Tumor Node Metastasis (TNM) stages or metastasis who might benefit from targeted LCN2 suppression to limit cancer spread (92). CEACAM5-targeted CAR T-cells7 have been developed to ComBat lung cancer (93). However, they are not very effective in fighting solid tumors (93). Currently, antibody drug conjugated (ADCs) immunotherapies are being developed to target various cancers including colorectal and gastric cancers (93). ADCs target receptors on the tumor surface, leading to the elimination of the tumor cell (93). SAR408701 is a promising candidate for treatments targeting CEACAM 5-positive cancers (94). Decary et al., found that SAR408701 has antiproliferative activity and significantly decreases tumor growth in cynomolgus monkeys (94). It has also been found in mouse models to lead to sustained tumor-free survival (94). Decary et al., suggested that the next step for this treatment is human clinical trials and hence is aligned with higher TNM stages where CEACAM5 expression is elevated. TFF3 has been found to be notably overexpressed in the later stages of CRC (95). AMPC, a small molecule inhibitor of TFF3 has emerged as a potential therapeutic option for colorectal cancer (95). TFF3 is known to activate ERK1/2, treatment using AMPC has decreased TFF3-mediated activation of Erk1/2 leading to decreased proliferation and decreased cell survival (95). Its synergistic effect with 5-fluorouracil further supports AMPC’s potential role in combination therapies, particularly for patients with advanced TNM stages. (95). TFF3 is overexpressed in colorectal cancer patients (96). TFF3 overexpression is more prominent in later-stage disease, therefore, it would be a better biomarker for more advanced TNM stages (96). Similarly to TFF3, CEACAM5 is overexpressed in later-stage disease as in the earlier stages CEACAM5 levels are too similar to those in healthy tissue (97) CEACAM5 has also been suggested to be more useful for detecting liver metastasis in CRC, suggesting it would be better at detecting cancers in more advanced TNM stages (98). In terms of clinical frameworks, both TFF3 and CEACAM5 exhibit overexpression primarily in later-stage disease, aligning them as potential biomarkers for higher TNM stages. CEACAM5 is also specifically associated with detecting liver metastasis in CRC (98), reinforcing its value in assessing metastatic spread. This suggests a dual role for these biomarkers, not only as therapeutic targets but also as indicators of disease stage, thereby informing prognosis and guiding treatment strategies in a stage-specific manner.

The validation results revealed the important roles of the observed proteins in the development of colorectal cancer (CRC) and offered good quantitative support for the prior findings. With a fold change of 3.5, TFF3 was confirmed to be the most important protein in the pathophysiology of colorectal cancer. Through pathways including PI3K/Akt and Src, its increased expression validates its function in increasing aggressive tumor behavior, boosting cell migration, and inducing the epithelial-mesenchymal transition (EMT). Similar to the previous findings, TFF1’s validation with a fold change of 2.8 confirmed its role in tumor cell invasiveness through EGFR signaling and G-protein-coupled receptor pathways. SELE’s validated role in blocking apoptosis via the PI3K/Akt/NFκB pathway was significantly consistent with its 2.5 fold change. This validation shows its functional significance in apoptosis resistance and tumor progression and confirms its potential as a biomarker of prediction for colorectal cancer. In the validation trials, AHCY displayed fold changes of 3.2 supporting its involvement in methylation processes that aid in the advancement of colorectal cancer. These results confirmed the previous evidence that it has a role in controlling the amounts of S-adenosylhomocysteine and S-adenosylmethionine, which are essential for epigenetic changes in tumor cells. LCN2, which has been verified with an unusual fold change of more than 20, offers compelling evidence of its function as a metastasis driver by stabilizing matrix metalloproteinase-9 (MMP9). Its significance in CRC pathogenesis is further supported by this remarkable confirmation, which emphasizes its role in extracellular matrix remodeling and tumor cell invasion. With a confirmed fold change of an average of 18, CEACAM5 is consistent with previous research showing that it plays a role in anoikis resistance and the activation of survival signaling pathways, including PI3K/Akt. Its importance is further shown by the validation results, which support its function in interfering with extracellular matrix-cell adhesion and promoting the development of secondary tumors. With a fold change of 2.5, Resistin (RETN) was confirmed to be associated with pro-inflammatory and carcinogenic signaling pathways, such as toll-like receptor-mediated ERK activation. Its role in tumor cell survival and advancement is confirmed by the validation results. When taken as a whole, the evidence of fold changes across these proteins not only demonstrates their steady elevation in colorectal cancer (CRC), but it also reinforces their biological significance in important pathways like metastasis, methylation regulation, apoptosis resistance, and EMT. The reliability and significance of these biomarkers in colorectal cancer (CRC) is confirmed by the high agreement between the initial findings and the validation results. TFF3, TFF1, SELE, AHCY, LCN2, CEACAM5, and RETN are consistently upregulated across a variety of datasets and analyzes, which supports their crucial roles in the underlying mechanisms of colorectal cancer progression. Their biological significance is confirmed by this congruence, which also emphasizes their potential as reliable biomarkers for prognostic and diagnostic applications. These proteins are useful in understanding the pathophysiology of colorectal cancer (CRC) because of their confirmed roles in important oncogenic processes, including methylation dysregulation, apoptosis resistance, epithelial-mesenchymal transition (EMT), and metastatic spread.

These biomarkers’ proven trustworthiness offers a strong foundation for creating prognosis devices, diagnostic tests, and individualized treatment plans. Additionally, incorporating them into medical processes may help detect colorectal cancer (CRC) earlier, increase the accuracy of treatment approaches, and benefit patients in general. By highlighting the importance of these indicators in furthering both research and clinical applications, this agreement between initial discovery and validation creates a crucial first step towards incorporating them into evidence-based CRC care techniques.

Our study reveals how biomolecules TFF3, LCN2, CEACAM5, SELE, RETN, TFF1, and AHCY are involved in CRC progression through proteogenomic pathways such as PI3k/Akt, EGFR, PLC/PKC. They promote invasion and metastasis suggesting new therapeutic targets and diagnostic markers. Understanding this molecular mechanism can enhance early detection and pave the way for more effective and personalized treatment approaches.

Further research is required to standardize the methodology in order to assess the stability of these proteins. The development of prediction models with benefits for clinical practice may be improved by the biological significance of stable protein selection. Some associations with colorectal cancer remain unexplored because proteomic data are not available for validation, even novel protein associations need to be found.

#### 4.4.1 Limitations of the study

Nevertheless, there are several limitations to this study. Limited datasets were available for validation. This affected the breadth of our investigation and the potential for generalization of our conclusions. Incompatibility of the discovery and validation datasets was seen. The mere recognition of patients with CRC, without considering comorbidities, introduces confounding variables that might have influenced the predicted accuracy of the model, since these additional ailments may have significantly changed the proteomic profile of certain samples. The substantial size of the UK Biobank sample, although beneficial, would not have completely mitigated the impact of these confounding comorbidities. A further challenge emerges due to an imbalanced distribution of samples in both the UK Biobank and *Bosch* et al. datasets. This might introduce bias in the training and assessment of the model, especially in the smaller *Bosch* et al. dataset, which had a relatively small total number of samples. Since different technologies like Olink and liquid chromatography coupled with tandem mass spectrometry (LC-MS) were used in quantification, the common proteins to validate were limited. There was also a lack of comprehensive demographic details like ethnicity in the validation dataset which restricts our ability to know how they impact our results and to ensure these results should be both representative and actionable across diverse populations. Also, regarding identifying the cohort, we could have explored in our analysis the time of diagnosis, as having received treatment would make a key variable to acknowledge. Finally, these 240 controls are not matched to the specific 269 as they come from the original matching between 9,890 diseased *vs* 9,890 controls.

#### 4.4.2 Future work

Given that our cohort counts with metabolite data, we plan to incorporate it into future analysis too. Also, to further validate our results, we want to focus on the experimental validation of the Fecal Immunochemical Test (FIT test), incorporating gene knockdown, biological pathways, and western blot analysis to assess the reliability and practical utility. This can ensure a thorough evaluation and understanding of our findings and their underlying mechanisms. Additionally, data validation across multiple datasets can be carried out with the integration of proteomics with transcriptomics, metabolomics, and other non-omics. Employing various machine learning models including stacking and deep neural algorithms can strengthen robustness and generalization can be possible.

## 5 Conclusion

The study has identified potential biomarkers that exhibit stability across various cases of colorectal cancer. These results offer valuable insight for identifying potential biomarkers in future proteomic studies with the goal of creating therapeutic strategies for patients with colorectal cancer. Nevertheless, further study is needed to look into the relationships and mechanistic properties of potential biomarkers.

## Data Availability

UK Biobank data access is indeed restricted: it is available exclusively through the UKB platform, contingent on an approved project plan, as outlined in UKB’s access policy. We confirm that all UKB data remains within the UKB infrastructure, and we have adhered to their policy by not depositing this data in external repositories.

## Glossary

AHCY: Adenosylhomocysteina
AUC: Area Under the ROC Curve
CART: Classification and regression trees
CEACAM5: Carcinoembryonic antigen-related cell adhesion molecule-5
CRC: Colorectal cancer
DEP: Differentially expressed proteins
FDR: False discovery rate
FOBT: Fecal occult blood test
FPR: False positive rate (FPR)
HPA: Human Protein Atlas
HPP: Human Proteome Project
KEGG: Kyoto Encyclopedia of Genes and Genomes
KNN: K-Nearest Neighbors
K-S test: Kolmogorov Smirnov Test
LASSO: Least Absolute Shrinkage and Selection Operator
LC-MS: Liquid chromatography-mass spectrometry
LCN2: Lipocalin 2
LightGBM: Light Gradient-Boosting Machine
NGS: Next-generation sequencing
NSAID: Non-steroidal anti-inflammatory drugs
PEA: Proximity Extension Assay
pQTL: Protein quantitative trait loci (pQTL) analysis
RETN: Resistin
SELE: Selectin E
SHAP: SHapley Additive exPlanations
SVM: Support vector machine
TFF1: Trefoil factor 1
Tff3: Trefoil factor 3
TPR: True positive rate
WGCNA: Weighted Gene Co-expression Network Analysis
XAI: Explainable artificial intelligence
XGBoost: eXtreme Gradient Boosting

## References

[1] Siegel R DeSantis C Jemal A. Colorectal cancer statistics, 2014. CA: A Cancer J Clin. (2014) 64:104–17. doi: 10.3322/caac.21220 24639052

[2] Ghoncheh M Mohammadian M Mohammadian-Hafshejani A Salehiniya H. The incidence and mortality of colorectal cancer and its relationship with the human development index in asia. Ann Global Health. (2017) 82(4):726. doi: 10.1016/j.aogh.2016.10.004 28283123

[3] Osterman E Hammarström K Imam I Osterlund E Sjöblom T Glimelius B. Recurrence risk after radical colorectal cancer surgery–less than before, but how high is it? Cancers. (2020) 12:3308. doi: 10.3390/cancers12113308 33182510 PMC7696064

[4] Kwak EL Chung DC. Hereditary colorectal cancer syndromes: an overview. Clin Colorectal Cancer. (2007) 6:340–4. doi: 10.3816/CCC.2007.n.002 17311698

[5] Ruder EH Laiyemo AO Graubard BI Hollenbeck AR Schatzkin A Cross AJ. Non-steroidal anti-inflammatory drugs and colorectal cancer risk in a large, prospective cohort. Am J Gastroenterology. (2011) 106(7):1340–50. doi: 10.1038/ajg.2011.38 PMC3183504 21407185

[6] Parry WH Martorano F Cotton EK. Management of life-threatening asthma with intravenous isoproterenol infusions. Am J Dis Children. (1976) 130:39–42. doi: 10.1001/archpedi.1976.02120020041006 2007

[7] Fan J Peng Z Zhou C Qiu G Tang H Sun Y. Gene-expression profiling in chinese patients with colon cancer by coupling experimental and bioinformatic genomewide gene-expression analyses: identification and validation of IFITM3 as a biomarker of early colon carcinogenesis. Cancer. (2008) 113(2):266–75. doi: 10.1002/cncr.23551 18470904

[8] Li C Wang H Lin F Li H Wen T Qian H. Bioinformatic exploration of MTA1-regulated gene networks in colon cancer. Front Med. (2016) 10(2):178–82. doi: 10.1007/s11684-016-0442-2 27052252

[9] Ritchie K Carrière I Ritchie CW Berr C Artero S Ancelin M-L. Designing prevention programmes to reduce incidence of dementia: prospective cohort study of modifiable risk factors. BMJ. (2010) 341:c3885. 20688841 10.1136/bmj.c3885 PMC2917002

[10] Bravo-Merodio L Acharjee A Russ D Bisht V Williams JA Tsaprouni LG. Translational biomarkers in the era of precision medicine. Adv Clin Chem. (2021) 102:191–232. doi: 10.1016/bs.acc.2020.08.002 34044910

[11] Liang Y Zhang C Dai D-Q. Identification of differentially expressed genes regulated by methylation in colon cancer based on bioinformatics analysis. World J Gastroenterol. (2019) 25:3392–407. doi: 10.3748/wjg.v25.i26.3392 PMC6639549 31341364

[12] Aziz F Acharjee A Williams JA Russ D Bravo-Merodio L Gkoutos GV. Biomarker prioritisation and power estimation using ensemble gene regulatory network inference. Int J Mol Sci. (2020) 21(21):7886. doi: 10.3390/ijms21217886 33114263 PMC7660606

[13] Charkoftaki G Thompson DC Golla JP Garcia-Milian R Lam TT Engel J. Integrated multi-omics approach reveals a role of ALDH1A1 in lipid metabolism in human colon cancer cells. Chemico-Biological Interactions. (2019) 304:88–96. doi: 10.1016/j.cbi.2019.02.030 30851239 PMC7988342

[14] Bisht V Nash K Xu Y Agarwal P Bosch S Gkoutos GV. Integration of the microbiome, metabolome and transcriptomics data identified novel metabolic pathway regulation in colorectal cancer. Int J Mol Sci. (2021) 22(11):5763. doi: 10.3390/ijms22115763 34071236 PMC8198673

[15] O’Dwyer D Ralton LD O’Shea A Murray GI. The proteomics of colorectal cancer: identification of a protein signature associated with prognosis. PloS One. (2011) 6:e27718. doi: 10.1371/journal.pone.0027718 22125622 PMC3220687

[16] Suhre K McCarthy MI Schwenk JM. Genetics meets proteomics: perspectives for large population-based studies. Nat Rev Genet. (2021) 22:19–37. doi: 10.1038/s41576-020-0268-2 32860016

[17] Anderson NL Anderson NG. The human plasma proteome. Mol Cell Proteomics. (2002) 1:845–67. doi: 10.1074/mcp.R200007-MCP200 12488461

[18] The NCI CPTAC. Proteogenomic characterization of human colon and rectal cancer. Nature. (2014) 513:382–7. doi: 10.1038/nature13438 PMC4249766 25043054

[19] Chen Y Hou W Zhong M Wu B. Comprehensive proteomic analysis of colon cancer tissue revealed the reason for the worse prognosis of right-sided colon cancer and mucinous colon cancer at the protein level. Curr Oncol. (2021) 28:3554–72. doi: 10.3390/curroncol28050305 PMC8482240 34590603

[20] Gong Y Liu Y Wang T Li Z Gao L Chen H. Age-associated proteomic signatures and potential clinically actionable targets of colorectal cancer. Mol Cell Proteomics. (2021) 20:100115. doi: 10.1016/j.mcpro.2021.100115 34129943 PMC8441843

[21] Kang S Na Y Joung SY Lee SI Oh SC Min BW. The significance of microsatellite instability in colorectal cancer after controlling for clinicopathological factors. Med (Baltimore). (2018) 97(9):e0019. doi: 10.1097/MD.0000000000010019 PMC5851768 29489646

[22] Mier PD van den Hurk JJ. Lysosomal hydrolases of the epidermis. 2. ester hydrolases. Br J Dermatol. (1975) 93:391–8. doi: 10.1111/j.1365-2133.1975.tb06512.x 31

[23] Mantione KJ Kream RM Kuzelová H Ptáček R Raboch J Samuel JM. Comparing bioinformatic gene expression profiling methods: microarray and RNA-seq. Med Sci Monitor Basic Res. (2014) 20:138–42. doi: 10.12659/MSMBR.892101 PMC4152252 25149683

[24] Metzker ML. Sequencing technologies — the next generation. Nat Rev Genet. (2010) 11:31–46. doi: 10.1038/nrg2626 19997069

[25] Kim H-Y Lee S-G Oh T-J Lim SR Kim S-H Lee HJ. Antiproliferative and apoptotic activity of *Chamaecyparis obtusa* leaf extract against the HCT116 human colorectal cancer cell line and investigation of the bioactive compound by gas chromatography-mass spectrometry-based metabolomics. Molecules. (2015) 20(10):18066–82. doi: 10.3390/molecules201018066 PMC6332506 26445036

[26] Dalal N Jalandra R Sharma M Prakash H Makharia GK Solanki PR. Omics technologies for improved diagnosis and treatment of colorectal cancer: Technical advancement and major perspectives. Biomedicine Pharmacotherapy. (2020) 131:110648. doi: 10.1016/j.biopha.2020.110648 33152902

[27] Onwuka S Bravo-Merodio L Gkoutos GV Acharjee A. Explainable AI-prioritized plasma and fecal metabolites in inflammatory bowel disease and their dietary associations. iScience. (2024) 27(7):110298. doi: 10.1016/j.isci.2024.110298 39040076 PMC11261406

[28] Tong D Tian Y Zhou T Ye Q Li J Ding K. Improving prediction performance of colon cancer prognosis based on the integration of clinical and multi-omics data. BMC Med Inf Decision Making. (2020) 20(1):22. doi: 10.1186/s12911-020-1043-1 PMC7006213 32033604

[29] Rao J Zhang W Li X Wang Y Liu H Chen F. Molecular characterization of advanced colorectal cancer using serum proteomics and metabolomics. Front Mol Biosciences. (2021) 8:687229. doi: 10.3389/fmolb.2021.687229 PMC8353147 34386520

[30] Ye S-B Cheng Y-K Li P-S Zhang L Zhang L-H Huang Y. High-throughput proteomics profiling-derived signature associated with chemotherapy response and survival for stage II/III colorectal cancer. NPJ Precis Oncol. (2023) 7:50. doi: 10.1038/s41698-023-00400-0 37258779 PMC10232411

[31] Saghapour E Kermani S Sehhati M. A novel feature ranking method for prediction of cancer stages using proteomics data. PloS One. (2017) 12:e0184203. doi: 10.1371/journal.pone.0184203 28934234 PMC5608217

[32] Pellino G Gallo G Pallante P Capasso R De Stefano A Maretto I. Noninvasive biomarkers of colorectal cancer: role in diagnosis and personalised treatment perspectives. Gastroenterol Res Practice. (2018) 2018:2397863. doi: 10.1155/2018/2397863 PMC6020538 30008744

[33] Shaukat A Levin TR. Current and future colorectal cancer screening strategies. Nat Rev Gastroenterol Hepatol. (2022) 19:521–31. doi: 10.1038/s41575-022-00612-y PMC9063618 35505243

[34] Schirripa M Lenz H-J. Biomarker in colorectal cancer. Cancer J. (2016) 22:156–64. doi: 10.1097/PPO.0000000000000190 PMC4955946 27341592

[35] Sun BB Chiou J Traylor M Benner C Hsu Y-H Richardson TG. Plasma proteomic associations with genetics and health in the UK biobank. Nature. (2023) 622(7982):329–38. doi: 10.1038/s41586-023-06592-6 PMC10567551 37794186

[36] Bosch S Acharjee A Quraishi MN Bijnsdorp IV Rojas P Bakkali A. Integration of stool microbiota, proteome and amino acid profiles to discriminate patients with adenomas and colorectal cancer. Gut Microbes. (2022) 14(1):2139979. doi: 10.1080/19490976.2022.2139979 36369736 PMC9662191

[37] Agarwal P Wicklow BA Dart AB Hizon NA Sellers EAC McGavock JM. Integrative analysis reveals novel associations between DNA methylation and the serum metabolome of adolescents with type 2 diabetes: a cross-sectional study. Front Endocrinology. (2022) 13:934706. doi: 10.3389/fendo.2022.934706 PMC9593237 36303872

[38] Ghorbani H. Mahalanobis distance and its application for detecting multivariate outliers. Facta Universitatis Series: Mathematics Inf. (2019) 34(3):583–93. doi: 10.22190/FUMI1903583G

[39] Chen T Guestrin C. XGBoost: A scalable tree boosting system. In: Proceedings of the 22nd ACM SIGKDD international conference on knowledge discovery and data mining. San Francisco, California: ACM (2016). p. 785–94. doi: 10.1145/2939672.2939785

[40] Ke G Meng Q Finley T Wang T Chen W Ma W. LightGBM: A highly efficient gradient boosting decision tree. In: Guyon I von Luxburg U Bengio S Wallach H Fergus R Vishwanathan S Garnett R, editors. Advances in neural information processing systems. Red Hook, NY: Curran Associates, Inc (2017). p. 3146–54.

[41] Tibshirani R. Regression shrinkage and selection *via* the lasso. J R Stat Soc. (1996) 58:267–88. doi: 10.1111/j.2517-6161.1996.tb02080.x

[42] Ma X Xiao L Liu X Zhang Y Wang W Chen J. Feature selection using LASSO regression for prognostic biomarkers in colorectal cancer. BMC Cancer. (2020) 20:1–11.

[43] Woźniacki A Książek W Mrowczyk P. A novel approach for predicting the survival of colorectal cancer patients using machine learning techniques and advanced parameter optimization methods. Cancers. (2024) 16(18):3205. doi: 10.3390/cancers16183205 39335174 PMC11430446

[44] Zhang Y Huang Y Xu M Zhuang J Zhou Z Zheng S. Pathomics-based machine learning models for predicting pathological complete response and prognosis in locally advanced rectal cancer patients post-neoadjuvant chemoradiotherapy: insights from two independent institutional studies. BMC Cancer. (2024) 24:1580. doi: 10.1186/s12885-024-13328-w 39725903 PMC11670422

[45] Lundberg SM Lee SI. A unified approach to interpreting model predictions. Adv Neural Inf Process Syst. (2017) 30:4765–74. doi: 10.48550/arXiv.1705.07874

[46] Shi K Lin W Zhao X-M. Identifying molecular biomarkers for diseases with machine learning based on integrative omics. IEEE/ACM Trans Comput Biol Bioinf. (2021) 18:2514–25. doi: 10.1109/TCBB.2020.2986387 32305934

[47] Davis J Goadrich M. The relationship between precision-recall and ROC curves. In: Proceedings of the 23rd international conference on machine learning. Pittsburgh, Pennsylvania: ACM Press (2006). p. 233–40. doi: 10.1145/1143844.1143874

[48] Hand DJ. Assessing the performance of classification methods. Int Stat Rev. (2012) 80:400–14. doi: 10.1111/j.1751-5823.2012.00183.x

[49] Sokolova M Japkowicz N Szpakowicz S. Beyond accuracy, f-score and ROC: A family of discriminant measures for performance evaluation. In: Sattar A Kang B, editors. AI 2006: Advances in artificial intelligence. Springer Berlin Heidelberg Berlin, Heidelberg (2006). p. 1015–21. p. 4304.

[50] Dutschmann T-M Kinzel L Ter Laak A Baumann K. Large-scale evaluation of k-fold cross-validation ensembles for uncertainty estimation. J Cheminform. (2023) 15:49. doi: 10.1186/s13321-023-00709-9 37118768 PMC10142532

[51] Gunning D Aha DW. DARPA’s explainable artificial intelligence program. AI Mag. (2019) 40:44–58. doi: 10.1609/aimag.v40i2.2850

[52] Benjamini Y Hochberg Y. Controlling the false discovery rate: A practical and powerful approach to multiple testing. J R Stat Soc Ser B: Stat Method. (1995) 57:289–300. doi: 10.1111/j.2517-6161.1995.tb02031.x

[53] Isaac O Thiemer K. Biochemical studies on camomile components/III. *In vitro* studies about the antipeptic activity of (–)-alpha-bisabolol (author’s transl). Arzneimittelforschung. (1975) 25:1352–4. 21

[54] Szklarczyk D Gable AL Nastou KC Lyon D Kirsch R Pyysalo S. The STRING database in 2021: customizable protein–protein networks, and functional characterization of user-uploaded gene/measurement sets. Nucleic Acids Res. (2021) 49(D1):D605–12. doi: 10.1093/nar/gkaa1074 PMC7779004 33237311

[55] Thul PJ Lindskog C. The human protein atlas: A spatial map of the human proteome. Protein Sci. (2018) 27:233–44. doi: 10.1002/pro.v27.1 PMC5734309 28940711

[56] Langfelder P Horvath S. WGCNA: an r package for weighted correlation network analysis. BMC Bioinf. (2008) 9:559. doi: 10.1186/1471-2105-9-559 PMC2631488 19114008

[57] Bycroft C Freeman C Petkova D Band G Elliott LT Sharp K. The UK biobank resource with deep phenotyping and genomic data. Nature. (2018) 562(7726):203–9. doi: 10.1038/s41586-018-0579-z PMC6786975 30305743

[58] Mbatchou J Barnard L Backman J Marcketta A Kosmicki JA Ziyatdinov A. Computationally efficient whole-genome regression for quantitative and binary traits. Nat Genet. (2021) 53(7):1097–103. doi: 10.1038/s41588-021-00870-7 34017140

[59] Durinck S Spellman PT Birney E Huber W. Mapping identifiers for the integration of genomic datasets with the R/Bioconductor package biomaRt. Nat Protoc. (2009) 4:1184–91. doi: 10.1038/nprot.2009.97 PMC3159387 19617889

[60] Zhang Y Jenkins DF Solaiappan M Johnson WE. Alternative empirical bayes models for adjusting for batch effects in genomic studies. BMC Bioinf. (2018) 19. doi: 10.1186/s12859-018-2263-6 PMC6044013 30001694

[61] Yu Y Zhang N Mai Y Ren L Chen Q Cao Z. Correcting batch effects in large-scale multiomics studies using a reference-material-based ratio method. Genome Biol. (2023) 24:201. doi: 10.1186/s13059-023-03047-z 37674217 PMC10483871

[62] Leek JT Johnson WE Parker HS Jaffe AE Storey JD. The sva package for removing batch effects and other unwanted variation in high-throughput experiments. Bioinformatics. (2012) 28:882–3. doi: 10.1093/bioinformatics/bts034 PMC3307112 22257669

[63] Thomas J Klebanov A John S Miller LS Vegesna A Amdur RL. CEACAMS 1, 5, and 6 in disease and cancer: interactions with pathogens. Genes Cancer. (2023) 14(1):12–29. doi: 10.18632/genesandcancer.230 36741860 PMC9891707

[64] Huang S Chang S Liao T Yang M. Detection and clinical significance of CEACAM5 methylation in colorectal cancer patients. Cancer Sci. (2024) 115:270–82. doi: 10.1111/cas.v115.1 PMC10823287 37942534

[65] Picardo F Romanelli A Muinelo-Romay L Mazza T Fusilli C Parrella P. Diagnostic and prognostic value of B4GALT1 hypermethylation and its clinical significance as a novel circulating cell-free DNA biomarker in colorectal cancer. Cancers (Basel). (2019) 11(10):1598. doi: 10.3390/cancers11101598 31635093 PMC6826707

[66] Vande Voorde J Steven RT Najumudeen AK Ford CA Dexter A Gonzalez-Fernandez A. Metabolic profiling stratifies colorectal cancer and reveals adenosylhomocysteinase as a therapeutic target. Nat Metab. (2023) 5:1303–18. doi: 10.1038/s42255-023-00857-0 PMC10447251 37580540

[67] Yusufu A Shayimu P Tuerdi R Fang C Wang F Wang H. TFF3 and TFF1 expression levels are elevated in colorectal cancer and promote the malignant behavior of colon cancer by activating the EMT process. Int J Oncol. (2019) 55(4):789–804. doi: 10.3892/ijo.2019.4854 31432157 PMC6741840

[68] Yusufu A Shayimu P Tuerdi R Fang C Wang F Wang H. TFF3 and TFF1 expression levels are elevated in colorectal cancer and promote the malignant behavior of colon cancer by activating the EMT process. Int J Oncol. (2019) 55(4):789–804. doi: 10.3892/ijo.2019.4854 31432157 PMC6741840

[69] Cui H-Y Wang S-J Song F Cheng X Nan G Zhao Y. CD147 receptor is essential for TFF3-mediated signaling regulating colorectal cancer progression. Signal Transduction Targeted Ther. (2021) 6(1):268. doi: 10.1038/s41392-021-00677-2 PMC8280106 34262017

[70] Santiago-Sánchez GS Pita-Grisanti V Quiñones-Díaz B Gumpper K Cruz-Monserrate Z Vivas-Mejía PE. Biological functions and therapeutic potential of lipocalin 2 in cancer. Int J Mol Sci. (2020) 21(12):4365. doi: 10.3390/ijms21124365 32575507 PMC7352275

[71] Živalj M JA VG Stijlemans B. Lipocalin-2: A nurturer of tumor progression and a novel candidate for targeted cancer therapy. Cancers. (2023) 15:5159. doi: 10.3390/cancers15215159 37958332 PMC10648573

[72] Yio X Zhang JY Babyatsky M Chen A Lin J Fan QX. Trefoil factor family-3 is associated with aggressive behavior of colon cancer cells. Clin Exp Metastasis. (2005) 22(2):157–65. doi: 10.1007/s10585-005-6615-z 16086236

[73] Rodrigues S Nguyen QD Faivre S Bruyneel E Thim L Westley B. Activation of cellular invasion by trefoil peptides and src is mediated by cyclooxygenase- and thromboxane A₂ receptor-dependent signaling pathways. FASEB J. (2001) 15(9):1517–28. doi: 10.1096/fj.00-0802com 11427483

[74] McLean MH Thomson AJ Murray GI Fyfe N Hold GL El-Omar EM. Expression of neutrophil gelatinase-associated lipocalin in colorectal neoplastic progression: a marker of malignant potential? Br J Cancer. (2013) 108(12):2537–41. doi: 10.1038/bjc.2013.264 PMC3694245 23736029

[75] Sun Y Yokoi K Li H Gao J Hu L Liu B. NGAL expression is elevated in both colorectal adenoma–carcinoma sequence and cancer progression and enhances tumorigenesis in xenograft mouse models. Clin Cancer Res. (2011) 17(13):4331–40. doi: 10.1158/1078-0432.CCR-11-0226 PMC3575684 21622717

[76] Yan L Borregaard N Kjeldsen L Moses MA. The high molecular weight urinary matrix metalloproteinase (MMP) activity is a complex of gelatinase B/MMP-9 and neutrophil gelatinase-associated lipocalin (NGAL). J Biol Chem. (2001) 276:37258–65. doi: 10.1074/jbc.M106089200 11486009

[77] Liebig B Brabletz T Staege MS Wulfänger J Riemann D Burdach S. Forced expression of n-TCF-1B in colon cancer derived cell lines is accompanied by the induction of CEACAM5/6 and mesothelin. Cancer Letters. (2005) 223(1):159–67. doi: 10.1016/j.canlet.2004.10.013 15890249

[78] Camacho-Leal P Stanners CP. The human carcinoembryonic antigen (CEA) GPI anchor mediates anoikis inhibition by inactivation of the intrinsic death pathway. Oncogene. (2008) 27:1545–53. doi: 10.1038/sj.onc.1210789 17891182

[79] Samara RN Laguinge LM Jessup JM. Carcinoembryonic antigen inhibits anoikis in colorectal carcinoma cells by interfering with trail-R2 (DR5) signaling. Cancer Res. (2007) 67:4774–82. doi: 10.1158/0008-5472.CAN-06-4315 17510406

[80] Rodrigues S Rodrigue CM Attoub S Fléjou JF Bruyneel E Bracke M. Induction of the adenoma-carcinoma progression and Cdc25A-b phosphatases by the trefoil factor TFF1 in human colon epithelial cells. Oncogene. (2006) 25(50):6628–36. doi: 10.1038/sj.onc.1209665 16715141

[81] Gout S Morin C Houle F Huot J. Death receptor-3, a new e-selectin counter- receptor that confers migration and survival advantages to colon carcinoma cells by triggering p38 and ERK MAPK activation. Cancer Res. (2006) 66:9117–24. doi: 10.1158/0008-5472.CAN-05-4605 16982754

[82] Porquet N Poirier A Houle F Pin A-L Gout S Tremblay P-L. Survival advantages conferred to colon cancer cells by e-selectin-induced activation of the PI3K-NFκB survival axis downstream of death receptor-3. BMC Cancer. (2011) 11:285. doi: 10.1186/1471-2407-11-285 21722370 PMC3177907

[83] Singh S Chouhan S Mohammad N Bhat MK. Resistin causes G1 arrest in colon cancer cells through upregulation of SOCS 3. FEBS Lett. (2017) 591:1371–82. doi: 10.1002/feb2.2017.591.issue-10 28417458

[84] Sudan SK Deshmukh SK Poosarla T Holliday NP Dyess DL Singh AP. Resistin: An inflammatory cytokine with multi-faceted roles in cancer. Biochim Biophys Acta (BBA) - Rev Cancer. (2020) 1874(2):188419. doi: 10.1016/j.bbcan.2020.188419 PMC8117252 32822824

[85] Li N Xiao H Shen J Qiao X Zhang F Zhang W. SELE gene as a characteristic prognostic biomarker of colorectal cancer. J Int Med Res. (2021) 49(4):3000605211004386. doi: 10.1177/03000605211004386 33845603 PMC8047093

[86] Feng M Feng J Chen W Wang W Wu X Zhang J. Lipocalin2 suppresses metastasis of colorectal cancer by attenuating NF-kB-dependent activation of snail and epithelial mesenchymal transition. Mol Cancer. (2016) 15. doi: 10.1186/s12943-016-0564-9 PMC5135816 27912767

[87] Gisina A Novikova S Kim Y Sidorov D Bykasov S Volchenko N. CEACAM5 overexpression is a reliable characteristic of CD133-positive colorectal cancer stem cells. Cancer Biomarkers. (2021) 32(1):85–98. doi: 10.3233/CBM-203187 34092615 PMC12500037

[88] Rompou AV Bletsa G Tsakogiannis D Theocharis S Vassiliu P Danias N. An updated review of resistin and colorectal cancer. Cureus. (2024) 16(7):e65403. doi: 10.7759/cureus.65403 39184804 PMC11344879

[89] Bech JM Terkelsen T Bartels AS Coscia F Doll S Zhao S. Proteomic profiling of colorectal adenomas identifies a predictive risk signature for development of metachronous advanced colorectal neoplasia. Gastroenterology. (2023) 165(1):121–132.e5. doi: 10.1053/j.gastro.2023.03.208 36966943

[90] Emami S Le Floch N Bruyneel E Thim L May F Westley B. Induction of scattering and cellular invasion by trefoil peptides in src- and RhoA-transformed kidney and colonic epithelial cells. FASEB J. (2001) 15(2):351–61. doi: 10.1096/fj.00-0355com 11156951

[91] Kim SL Shin MW Seo SY Kim SW. Lipocalin 2 potentially contributes to tumorigenesis from colitis *via* IL-6/STAT3/NF-kB signaling pathway. Biosc Rep. (2022) 42:BSR20212418. doi: 10.1042/BSR20212418 PMC9109459 35470375

[92] Nuntagowat C Leelawat K Tohtong R. NGAL knockdown by siRNA in human cholangiocarcinoma cells suppressed invasion by reducing NGAL/MMP-9 complex formation. Clin Exp Metast. (2010) 27:295–305. doi: 10.1007/s10585-010-9327-y 20373132

[93] Kim Y-J Li W Zhelev DV Mellors JW Dimitrov DS Baek D-S. Chimeric antigen receptor-t cells are effective against CEACAM5 expressing non-small cell lung cancer cells resistant to antibody-drug conjugates. Front Oncol. (2023) 13:1124039. doi: 10.3389/fonc.2023.1124039 36923424 PMC10010383

[94] Decary S Berne P-F Nicolazzi C Lefebvre A-M Dabdoubi T Cameron B. Preclinical activity of SAR408701: A novel anti-CEACAM5–maytansinoid antibody–drug conjugate for the treatment of CEACAM5-positive epithelial tumors. Clin Cancer Res. (2020) 26(24):6589–99. doi: 10.1158/1078-0432.CCR-19-4051 33046521

[95] Chen RM Chiou YS Chong QY Poh HM Tan TZ Zhang MY. Pharmacological inhibition of TFF3 enhances sensitivity of CMS4 colorectal carcinoma to 5-fluorouracil through inhibition of p44/42 MAPK. Int J Mol Sci. (2019) 20:6215. doi: 10.3390/ijms20246215 31835445 PMC6940926

[96] Li Q Wang K Su C Fang J. Serum trefoil factor 3 as a protein biomarker for the diagnosis of colorectal cancer. Technol Cancer Res Treat. (2017) 16:440–5. doi: 10.1177/1533034616674323 PMC5616070 27760866

[97] Goldstein MJ Mitchell EP. Carcinoembryonic antigen in the staging and follow- up of patients with colorectal cancer. Cancer Invest. (2005) 23:338–51. doi: 10.1081/cnv-58878 16100946

[98] Duffy MJ. Carcinoembryonic antigen as a marker for colorectal cancer: is it clinically useful? Clin Chem. (2001) 47:624–30. doi: 10.1093/clinchem/47.4.624 11274010

