# Supplementary material for: Machine learning-based identification of proteomic markers in colorectal cancer using UK Biobank data
Source: Front Oncol. 2025 Jan 7;14:1505675. doi: 10.3389/fonc.2024.1505675 (PMC11746037; doi:10.3389/fonc.2024.1505675)
Supplement: Supplementary file 2 [file DataSheet1.docx]

**Supplementary Tables**

**Table.S1** Diagnosis codes

| **Diagnosis Source** | **Diagnosis Code** |
| --- | --- |
| Cancer Register (ICD10)  (UKBB fields 40006) | C180,C181,C182,C183,C184,C185,C186,C187,C188,C189,C19,C20 |
| Cancer Register (ICD9)  (UKBB fields 40013) | 1530, 1532, 1533, 1534,1536,1537, 1539, 1540, 1541 |
| Death registry (ICD10)  (UKBB fields 40001) | C180,C181,C182,C183,C184,C185,C186,C187,C188,C189,C19,C20 |
| First inpatient diagnosis (ICD10)  (UKBB fields 41270) | C180,C181,C182,C183,C184,C185,C186,C187,C188,C189,C19,C20 |
| First inpatient diagnosis (ICD9)  (UKBB fields 41271) | 1530, 1532, 1533, 1534,1536,1537, 1539, 1540, 1541 |
| Self reported  (UKBB fields 20001) | 1020, 1022,1023 |

********UKBB:UK Biobank*

**Table.S2** Phase 1 hypertuning parameters

| **Hyperparameters** | **LASSO** | **XGBoost** | **LightGBM** |
| --- | --- | --- | --- |
| colsample_bytre e | - | 1.0 | 0.8 |
| learning_rate | - | 0.05 | 0.05 |
| 'max_depth | - | 5 | 5 |
| n_estimators | - | 100 | 31 |
| subsample | - | 0.8 | 0.8 |
| Alpha | 0.01 | - | - |

**Table.S3** Phase 2 hypertuning parameters

| **Hyperparameters** | **LASSO** | **XGBoost** | **LightGBM** |
| --- | --- | --- | --- |
| colsample_bytree | - | 0.8 | 0.8 |
| learning_rate | - | 0.05 | 0.01 |
| 'max_depth | - | 5 | 10 |
| n_estimators | - | 200 | 31 |
| subsample | - | 0.8 | 0.8 |
| Alpha | 0.01 | - | - |

**Table.S4** Performance metric scores of the UK Biobank and *Bosch et.al.* (Phase 1)

**Table.S5.** Performance metric scores of the UK Biobank and *Bosch et.al.* (Phase 2)

**Table.S6** Phase 1 confusion metrics

| **Confusion matrix** | **True positives** | **True negatives** | **False positives** | **False negatives** |
| --- | --- | --- | --- | --- |
| **UK Biobank** |  |  |  |  |
| XGBoost | 39 | 29 | 20 | 15 |
| LightGBM | 38 | 29 | 20 | 16 |
| LASSO | 36 | 28 | 21 | 18 |
| ***Bosch et.al.*** |  | | | |
| XGBoost | 13 | 0 | 20 | 0 |
| LightGBM | 13 | 0 | 20 | 0 |
| LASSO | 13 | 0 | 20 | 0 |

**Table.S7** Phase 2 confusion metrics

|  | **True positives** | **True negatives** | **False positives** | **False negatives** |
| --- | --- | --- | --- | --- |
| **UK Biobank** |  |  |  |  |
| XGBoost | 33 | 25 | 24 | 21 |
| LightGBM | 34 | 26 | 23 | 20 |
| LASSO | 34 | 29 | 20 | 20 |
| ***Bosch et.al.*** |  | | | |
| XGBoost | 9 | 12 | 8 | 4 |
| LightGBM | 4 | 14 | 6 | 9 |
| LASSO | 2 | 18 | 2 | 11 |

**Table.S8** Functions of 7 proteins in Colorectal Cancer

| **Proteins** | **Function in Colorectal Cancer** |
| --- | --- |
| TFF3* | Proliferation, migration, and invasiveness of HT29 cells (67). |
| LCN2* | Enzymatic activity of matrix metalloprotease-9 causing metastasis (70). |
| CEACAM5* | Metastasis of tumor cells (94). |
| TFF1* | Activates signaling pathways (73). |
| SELE* | Activated the phosphoinositide 3-kinase (PI3K)/Akt pathway (85) |
| RETN* | Involved ERK signaling (83)(84) |
| AHCY* | Catalyzes the hydrolysis of S-adenosylhomocysteine (66). |

**Legend:**

**Tff3:Trefoil factor 3*

**LCN2:Lipocalin 2*

**CEACAM5:carcinoembryonic antigen-related cell adhesion molecule-5*

**TFF1:*Trefoil factor 1

*SELE:Selectin E

*RETN:*Resistin*

**AHCY:Adenosylhomocysteinase*

**Supplementary figures**

**A**

**B**


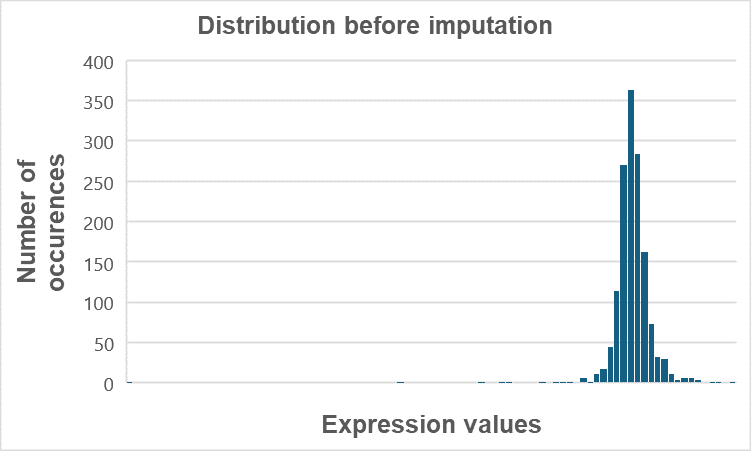

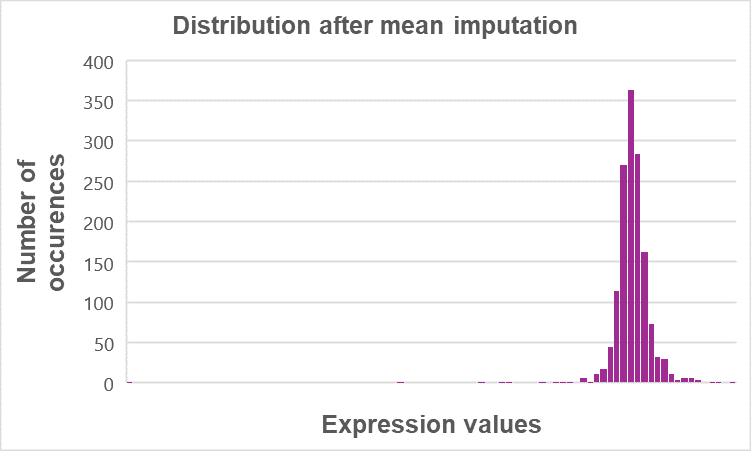


**D**

**C**


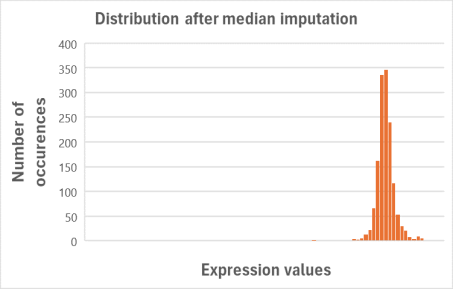

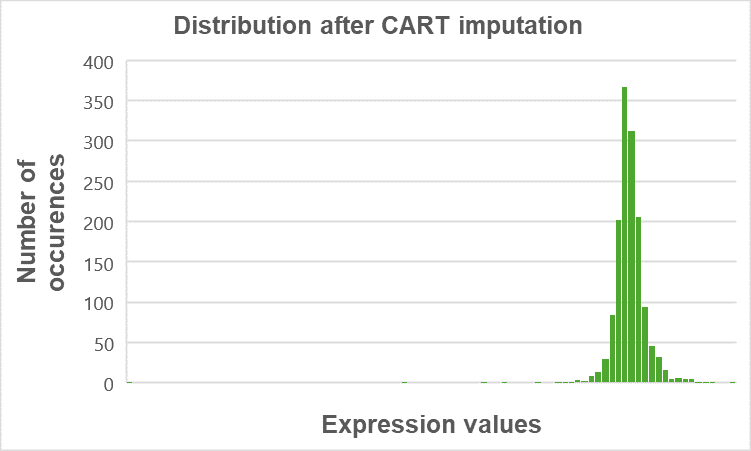


**E**


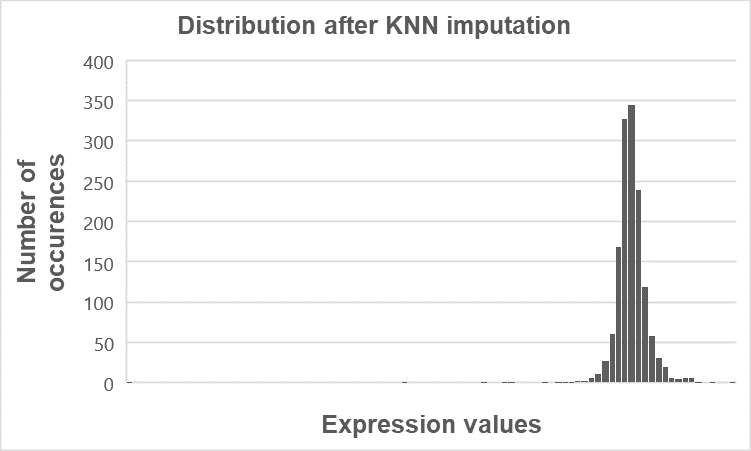


**Fig.S1.** Decision of missing value imputation.

**A** Distribution before imputation (p value (K-S test)=0.08). **B** Distribution after mean imputation (p value (K-S test)=0.08). **C** Distribution after median imputation (p value (K-S test)=0.17). **D** Distribution after CART* imputation (p value (K-S test)=0.30). **E** Distribution after KNN* imputation (p value (K-S test)=0.45).


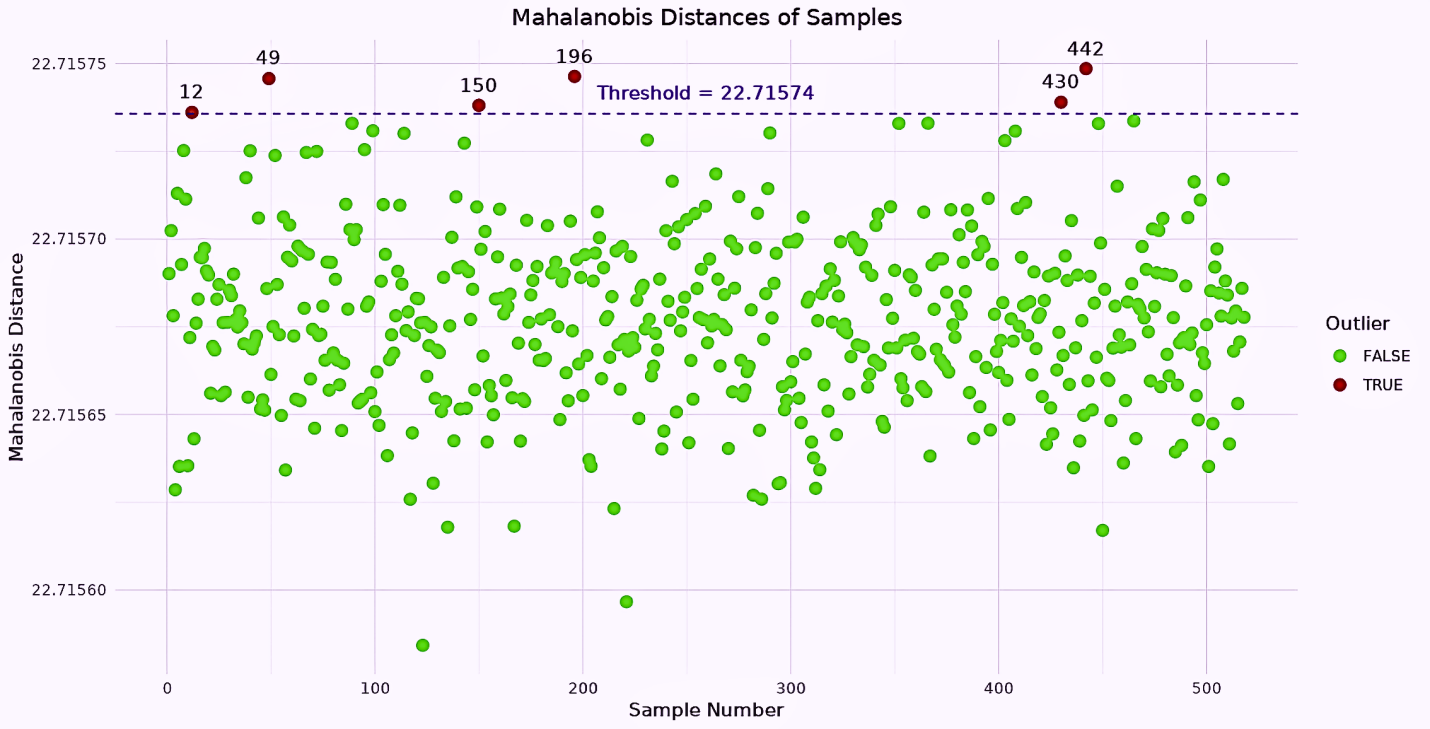


**Fig.S2** Mahalanobis distance of samples

Identification of the outliers in the UK Biobank dataset


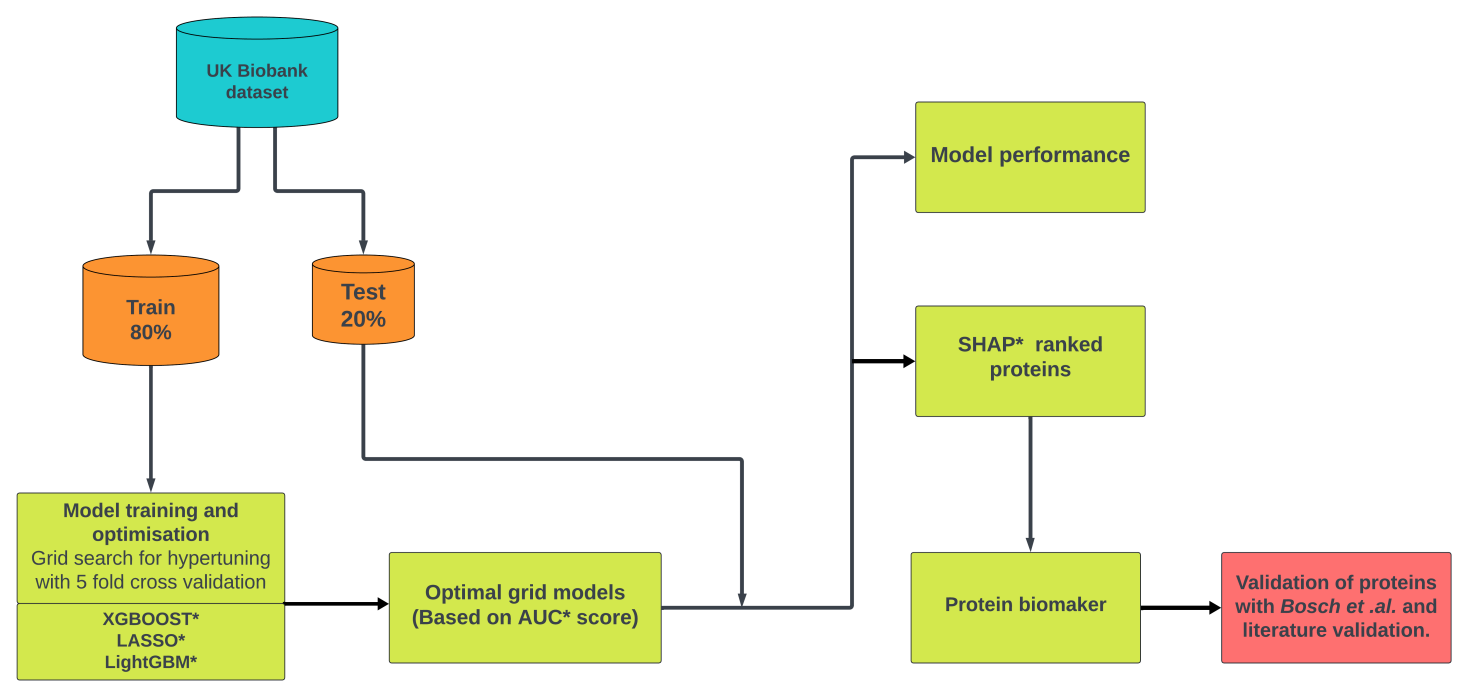


**Repeat 50 times**

**Fig.S3** Schematic diagram of the machine learning pipeline for protein biomarker analysis

**Legend**:
*XGBoost*: eXtreme Gradient Boosting
LASSO*: Least Absolute Shrinkage and Selection Operator
LightGBM*: Light Gradient-Boosting
SHAP*:SHapley Additive exPlanations
AUC*:Area under the ROC Curve*


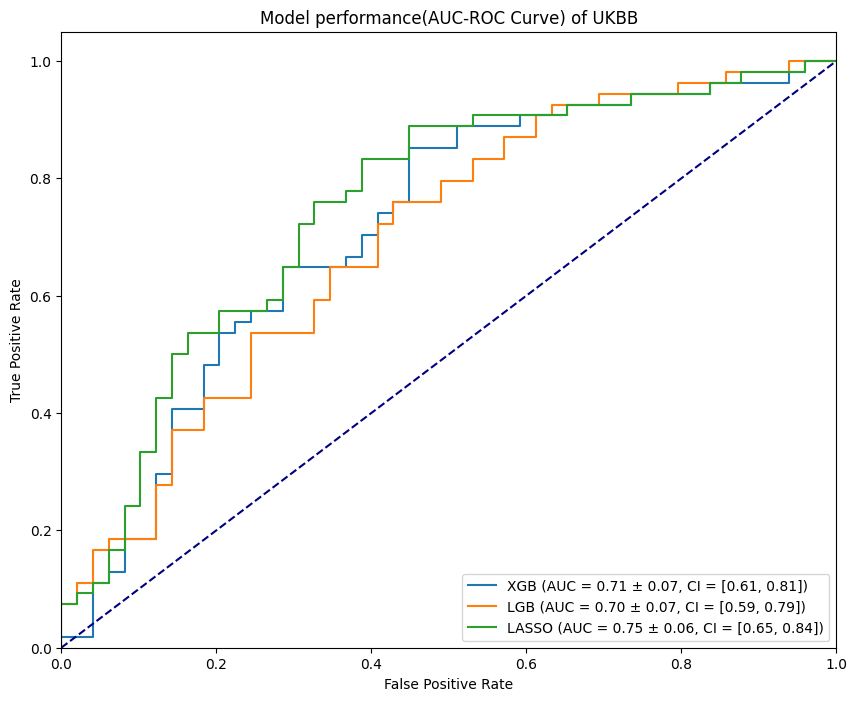

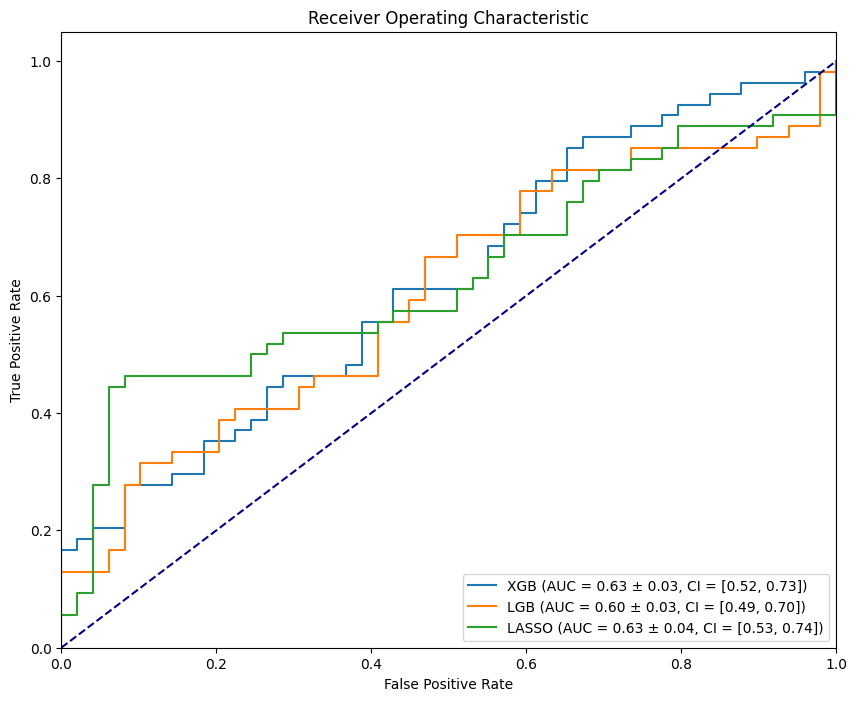


**B**

**A**

**Fig.S4** Model performance of UK Biobank

**A** Model performance (AUC-ROC) OF UK Biobank (Phase 1). **B** Model performance (AUC-ROC) of UK Biobank (Phase 2)


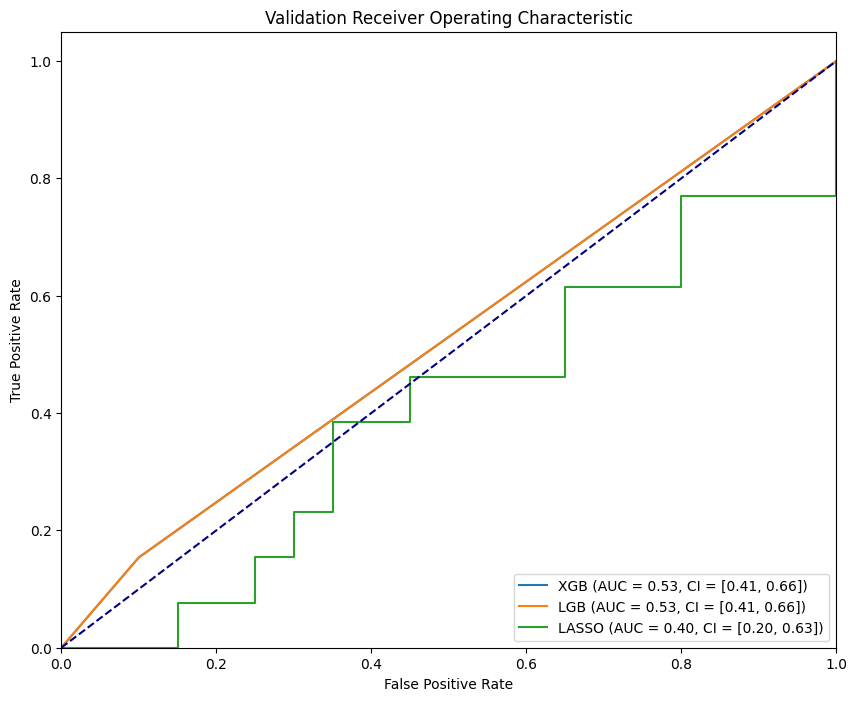


**B**

**A**


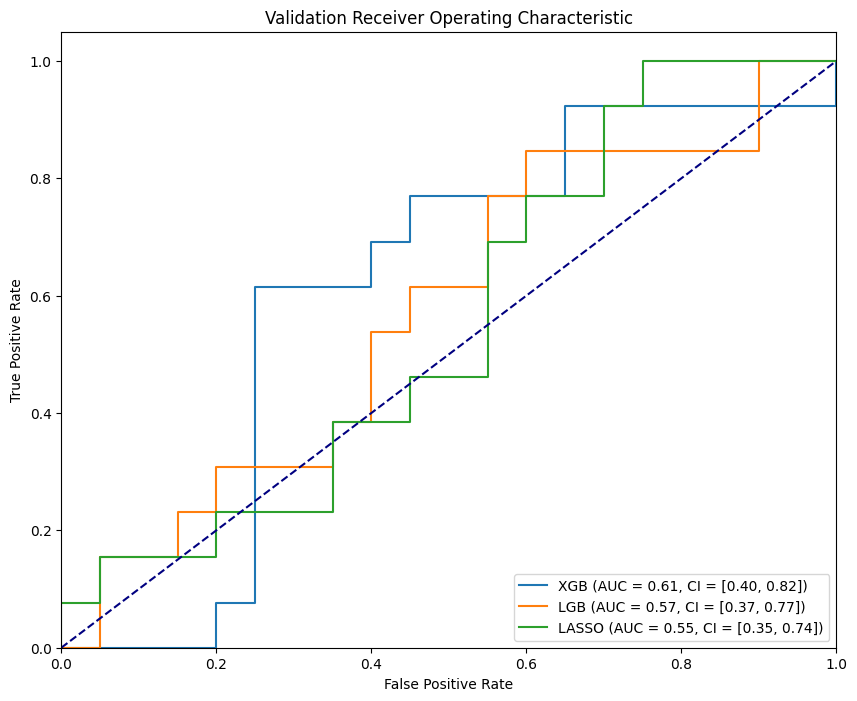


**Fig.S5** Model performance of *Bosch et.al.*

**A** Model performance (AUC-ROC) of *Bosch et.al*. (Phase 1). **B** Model performance (AUC-ROC) of *Bosch et.al.* (Phase 2 )


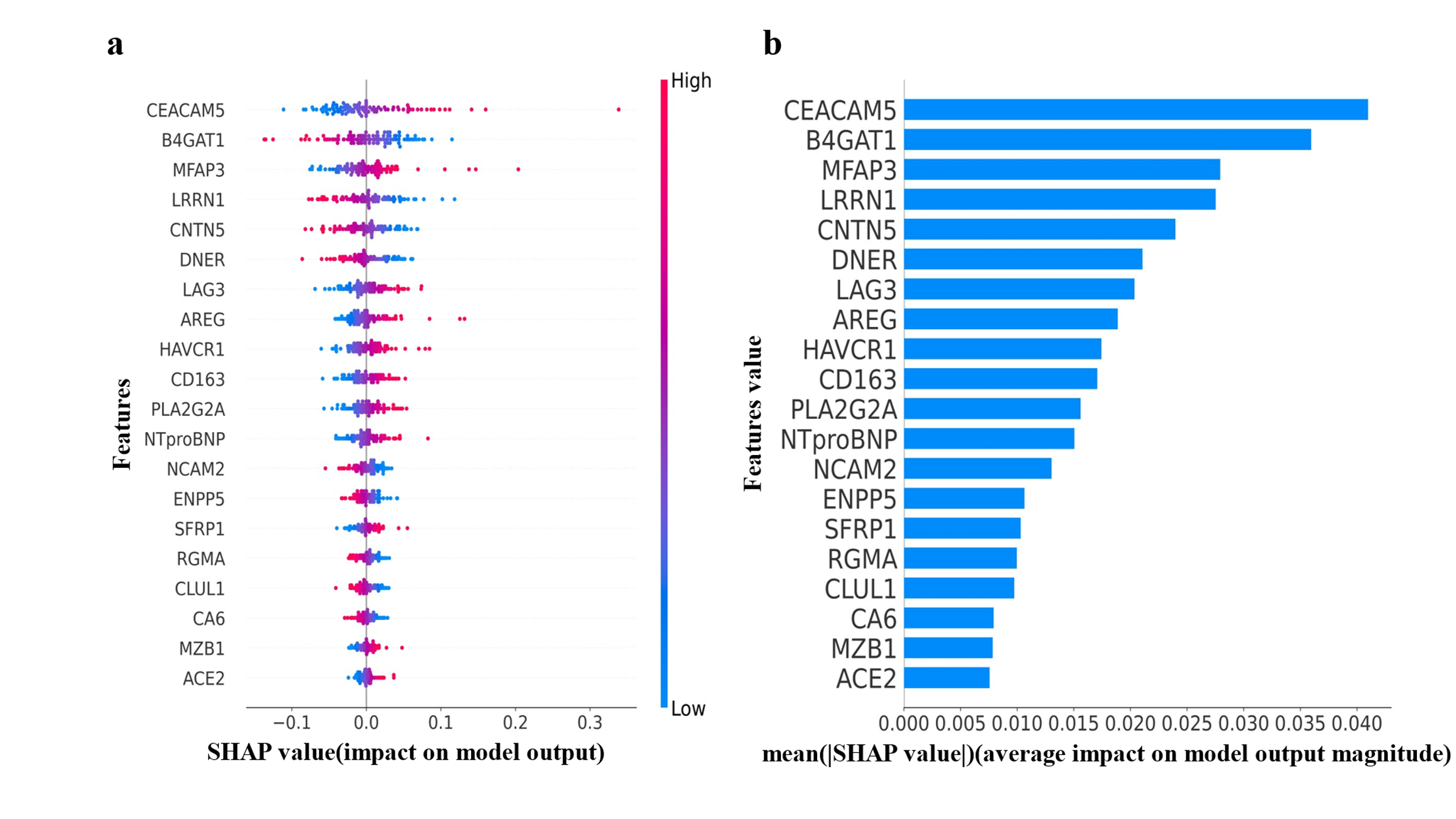


**A**

**B**

**C**

**Feature value**

**Features**


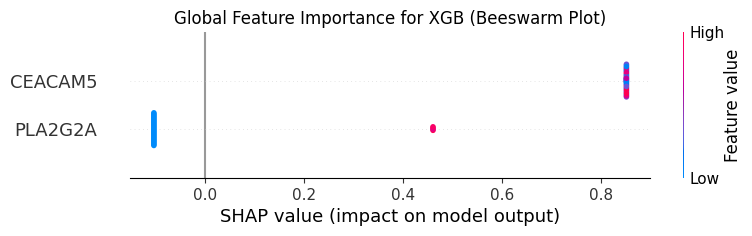


**SHAP value (impact on model output)**

**Features**


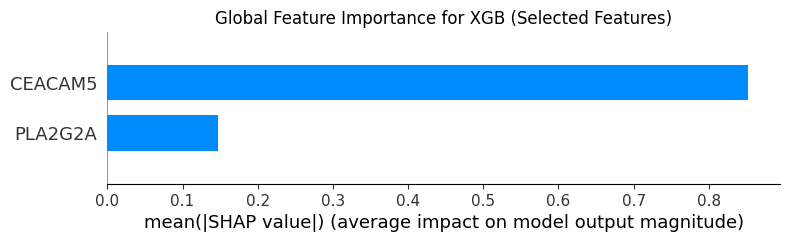


**D**

**mean (|SHAP value|)(average impact on model output magnitude)**

**Fig.S6** The figure illustrates the ranked SHAP summary plots of model predictions.

**A** UK Biobank - Local importance (LASSO-Phase1). **B** UK Biobank-Global importance (LASSO-Phase1).**C** *Bosch et.al.* - Local importance (XGBoost-Phase1). **D** *Bosch et.al*.-Global importance (XGBoost-Phase1).The features displayed are the top 20 proteins of each model, as determined by their SHAP values.The samples are shown as coloured dots in the local importance summary plot for SHAP values, the colour of each dot corresponds to its value for that feature. Positive SHAP values have a positive effect on the model and direct the algorithm to predict the positive class, and vice versa.In the global importance summary plot for mean absolute SHAP values higher rank features are associated with more samples having SHAP values.

**A**


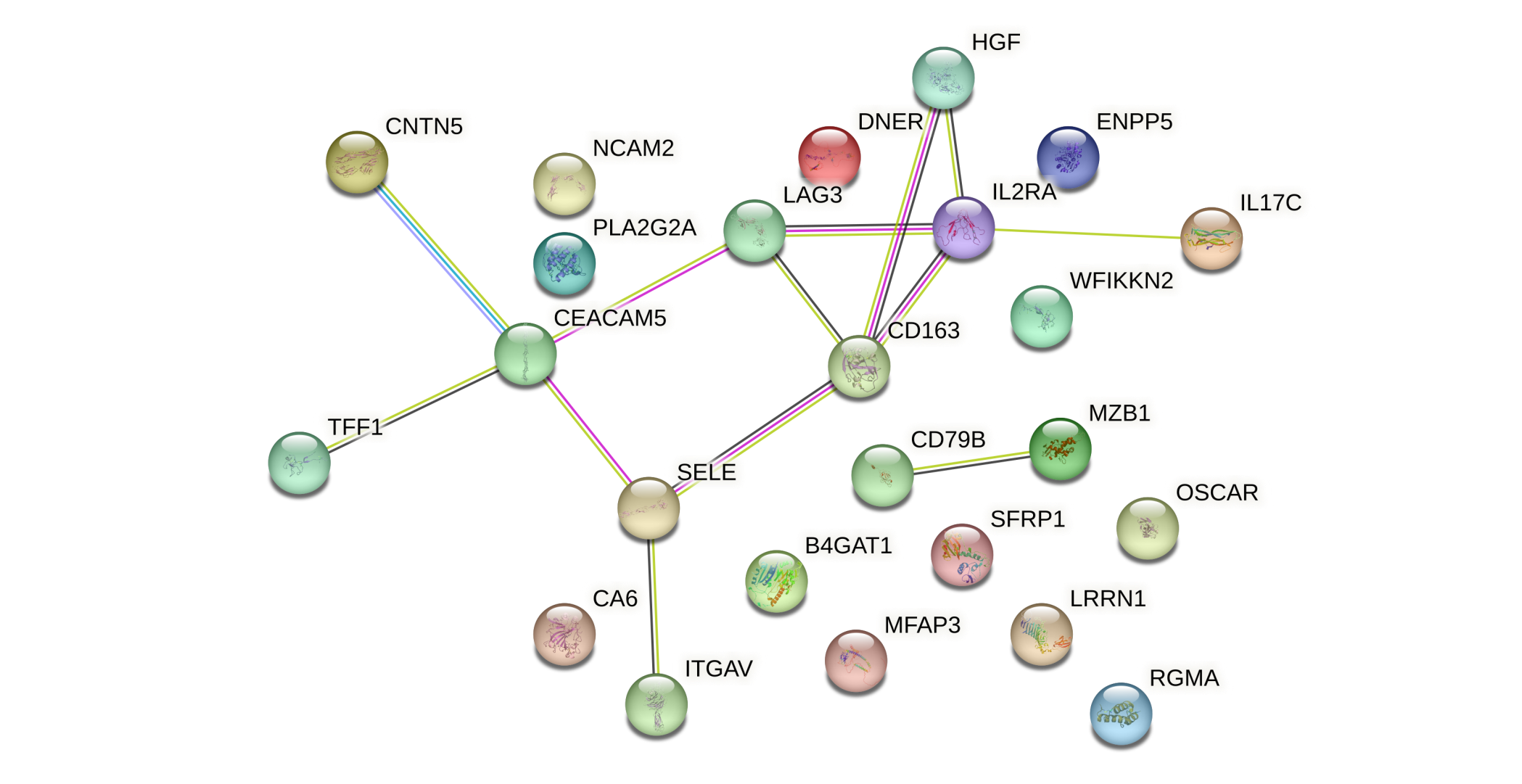


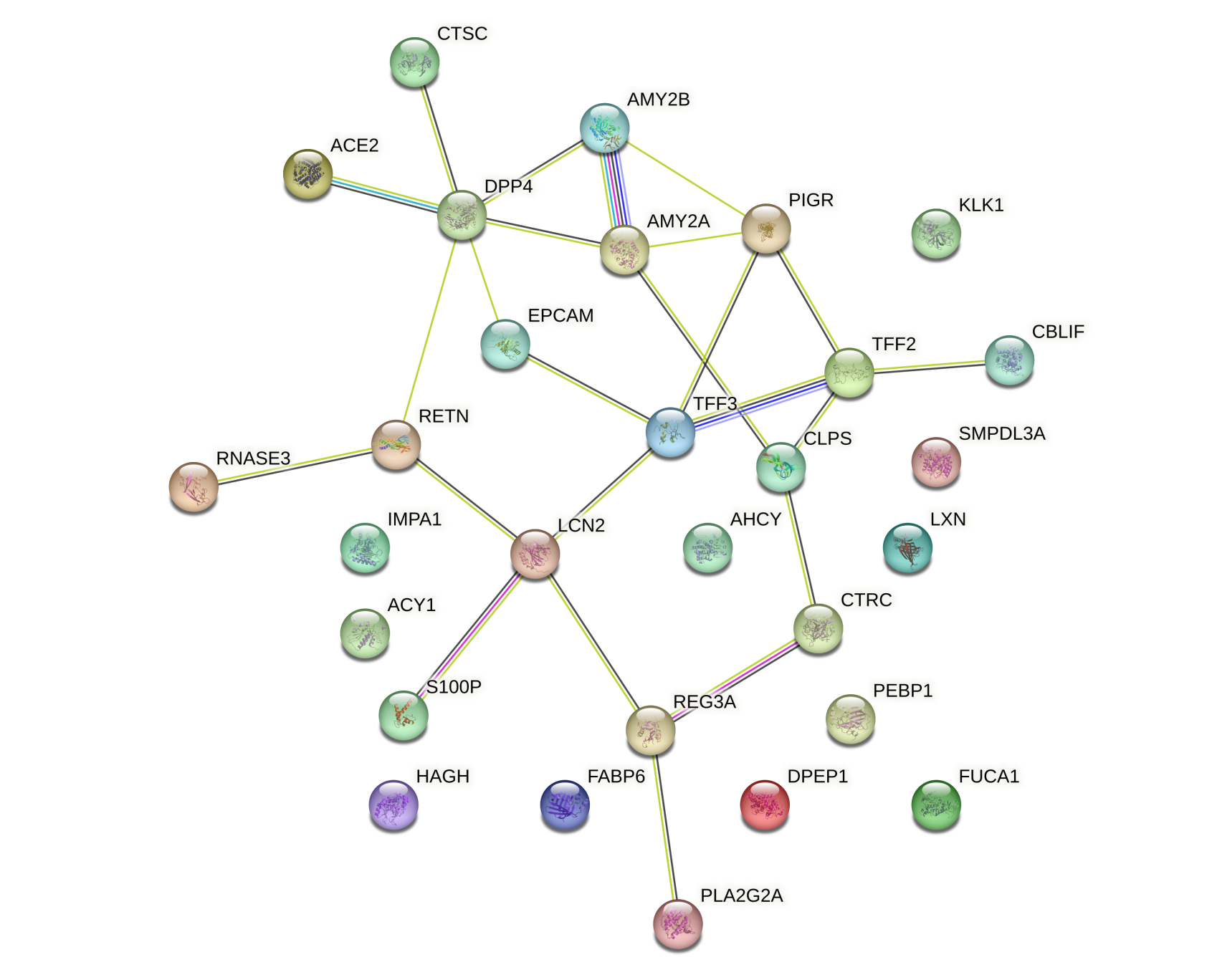


**B**


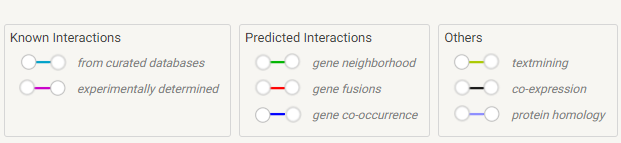


**Fig.S7 STRING protein-protein interaction network**

**A** STRING protein-protein interaction network of the 25 proteins. **B** STRING protein-protein interaction network of the 29 proteins

Nodes represent individual proteins. Edges represent the interactions between these proteins. Light blue lines represent interactions that have been confirmed and documented in curated biological databases. Pink lines indicate interactions that have been observed in laboratory experiments. Green lines represent interactions predicted based on genes that are located in close proximity on the chromosome. Red lines show predicted interactions where two or more genes have fused in certain species, suggesting functional relationships. Blue lines indicate interactions predicted based on the tendency of genes to co-occur across different species, implying evolutionary conservation of functional associations. Yellow lines derived from associations in scientific literature, where co-mentions of genes or proteins suggest potential interactions. Black lines show interactions between proteins with similar expression patterns across different conditions, tissues, or cell types. Purple lines indicate interactions between proteins with sequence similarities, suggesting functional or evolutionary relationships.

**Comparison of TFF3 expression between case and control**

**A**

**4**

**3**

**2**

**1**

**0**

**-1**


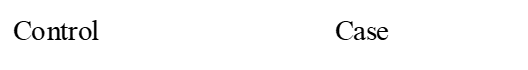


**B**

**Comparison of LCN2 expression between case and control**


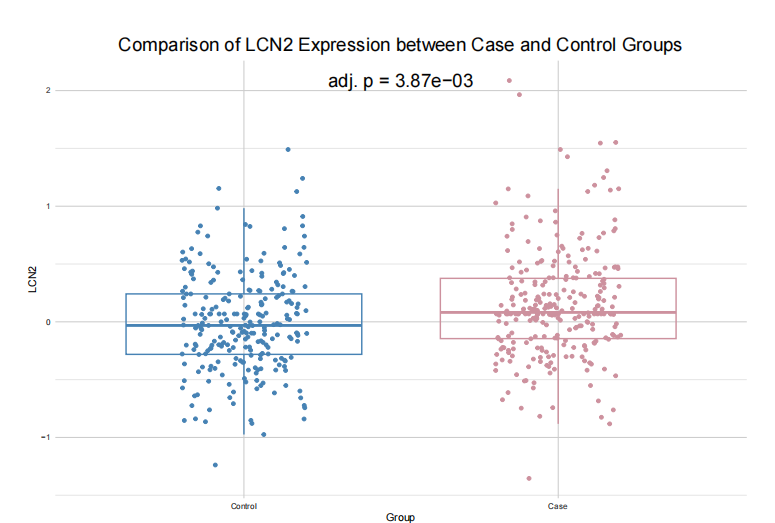


**2**

**1**

**0**

**-1**


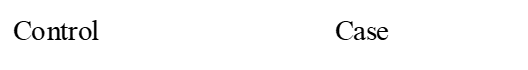


**Comparison of CEACAM5 expression between case and control**

**C**

**4**

**2**

**0**

**-2**


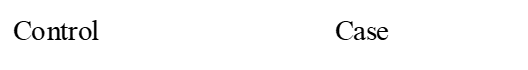


**D**

**Comparison of TFF1 expression between case and control**

**4**

**2**

**0**

**-2**


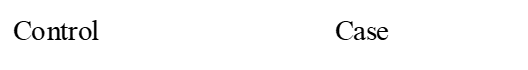


**Comparison of SELE expression between case and control**

**E**

**1**

**0**

**-1**


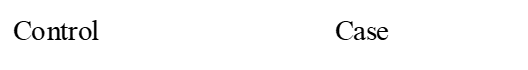


**F**

**Comparison of RETN expression between case and control**

**2**

**1**

**0**

**-1**

**-2**


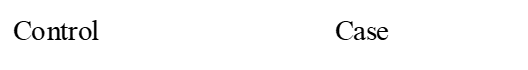


**Comparison of AHCY expression between case and control**

**3**

**2**

**1**

**0**

**-1**

**-2**

**G**

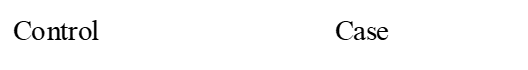


**Fig.S8 Comparison of seven proteins between case and control groups**

**A** Comparison of TFF3. **B** Comparison of LCN2. **C** Comparison of CEACAM5. **D** Comparison of TFF1. **E** Comparison of SELE. **F** Comparison of RETN. **G** Comparison of AHCY

**B**

**A**


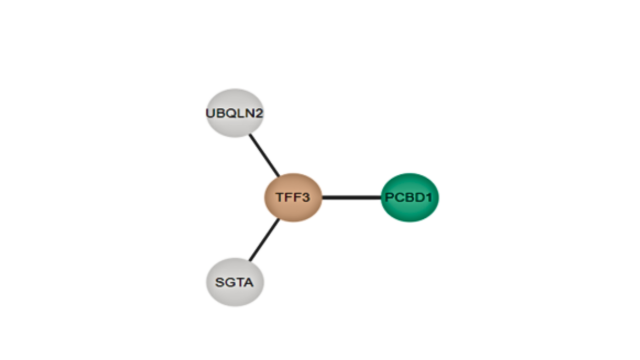

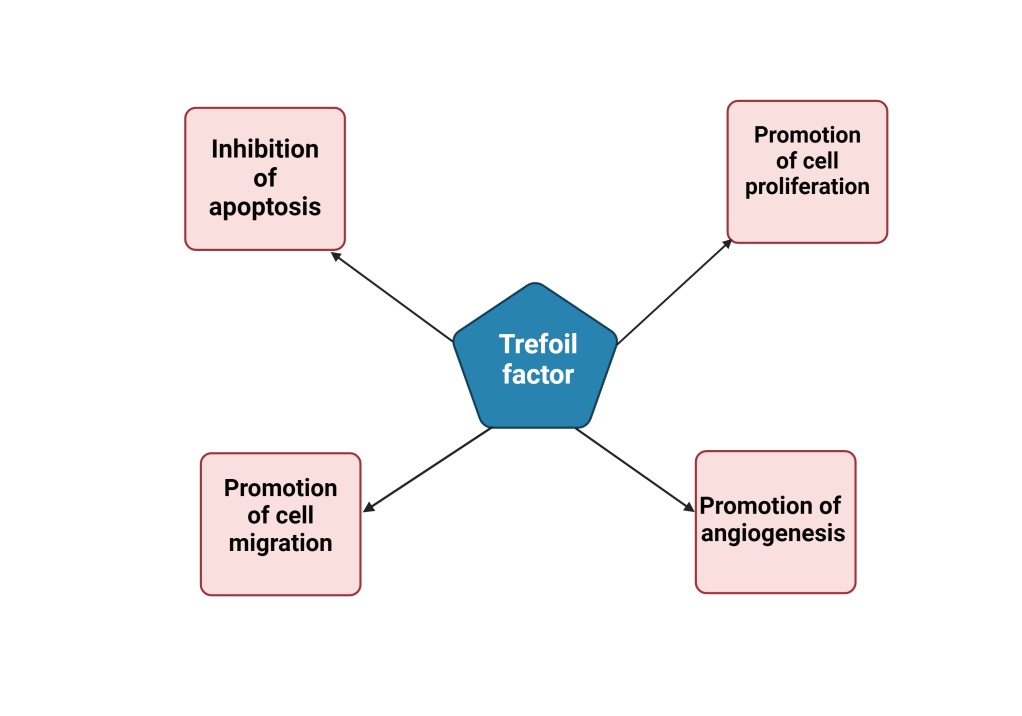


**Fig.S9** Significance of TFF3 in literature

**A** TFF3 interaction with other protein clas**s. B** Functions of TFF3 in colorectal cancer


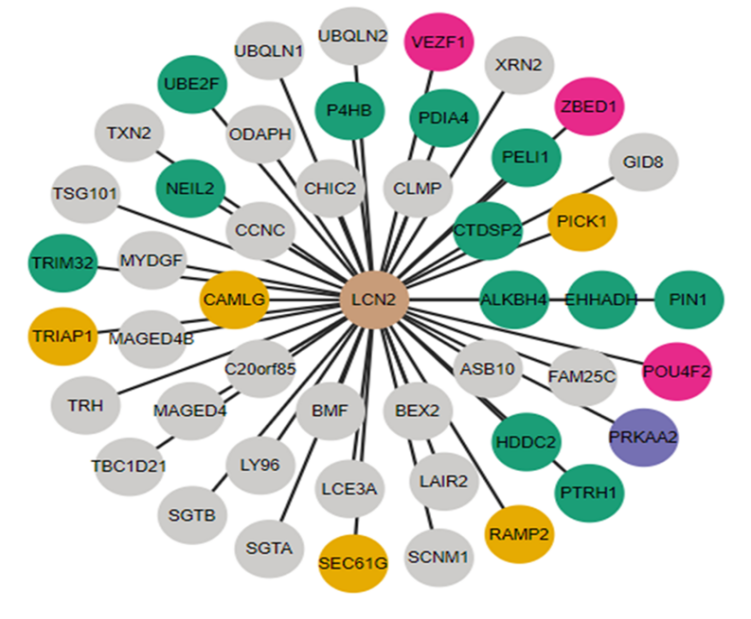

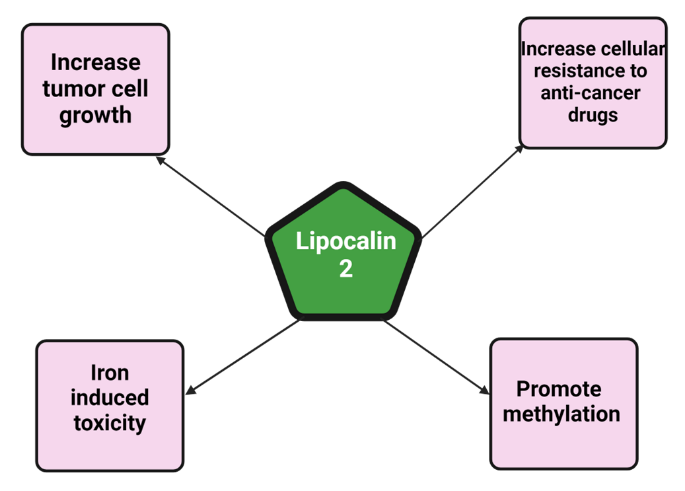


**B**

**A**

**Fig.10** Significance of LNC2 in literature

**A** LCN2 interaction with other proteins classes. **B** Functions of LCN2 in colorectal cancer


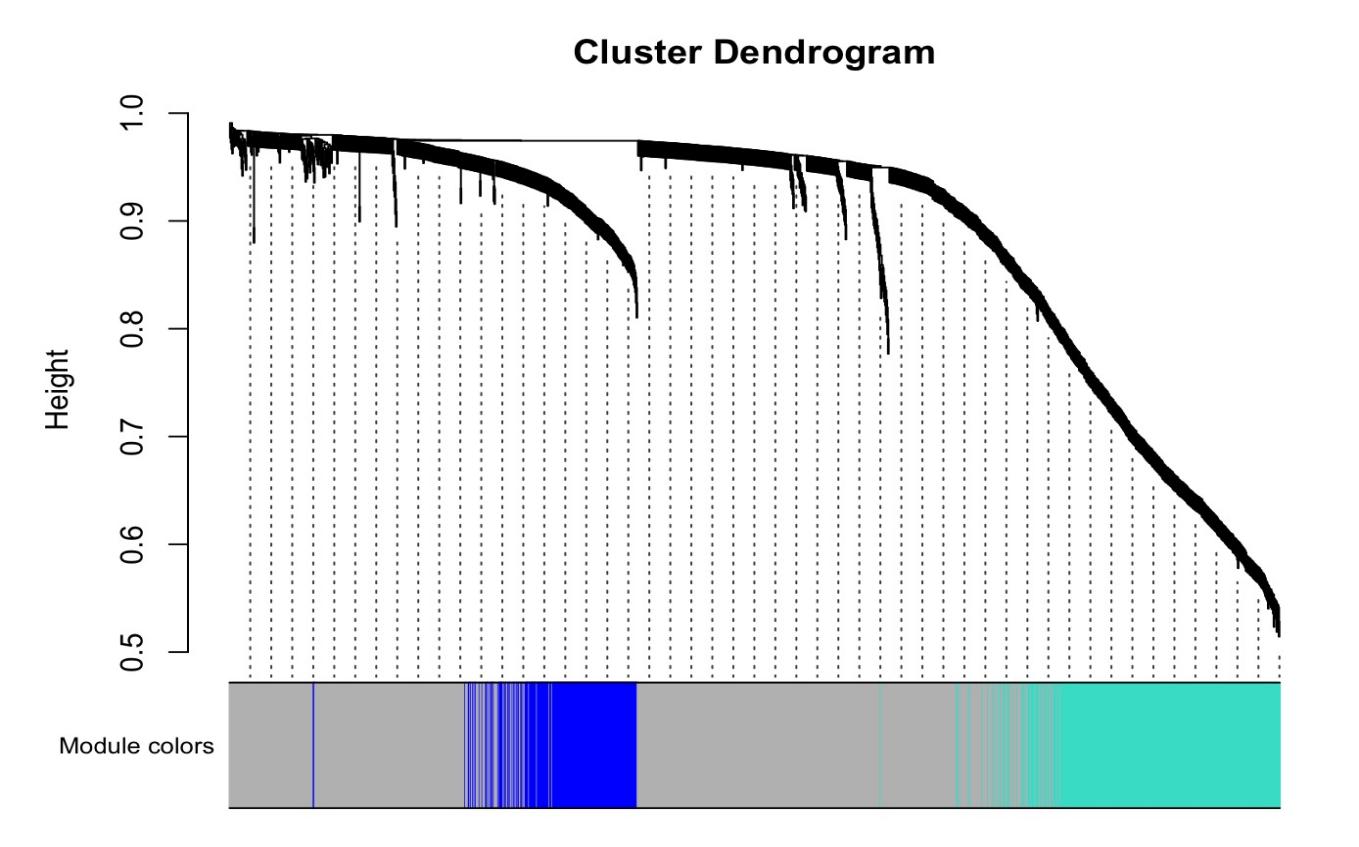


**Fig.S11** Module identification.

The cluster dendrogram represents a visual representation of the hierarchical clustering of genes based on their expression profiles. The height on the y-axis reflects the degree of dissimilarity between the genes. In the dendrogram, distinct clusters of genes are merged, and the corresponding module colours are displayed underneath.Two distinct modules, MEBlue and METurquoise, were identified and are represented by branches in blue and turquoise, respectively. Genes not grouped into these modules are assigned to the Gray module, indicating that they do not co-cluster with any of the defined modules. The MEBlue and METurquoise modules represent gene groups with similar expression patterns, reflecting shared biological functions and regulatory mechanisms.

**B**

**A**


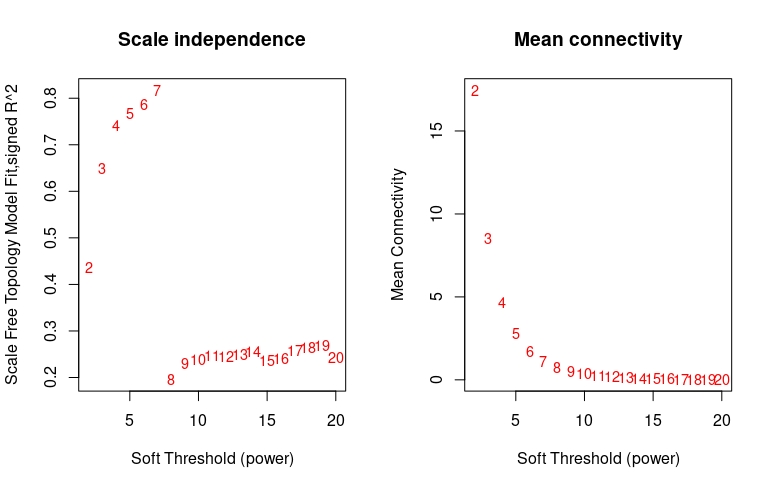


**Fig.S12** Assessment of Scale-Free Topology for UK Biobank dataset.

The figure displays the evaluation of the scale-free topology model fit and mean connectivity for a gene expression dataset from the UK Biobank. The X-axis represents the soft-thresholding power (β), while the Y-axis shows the signed R² value for the scale-free topology model fit and the mean connectivity. Although the **B** mean connectivity values were within expected ranges**, A** the signed R² values were consistently low, indicating a poor fit to the scale-free topology model. This suggested that the dataset did not exhibit the ideal scale-free network characteristics required for robust co-expression analysis. Consequently, due to the inadequate fit and overall unsuitability for WGCNA, no further analyses were conducted, and no modules were identified for this dataset.

 
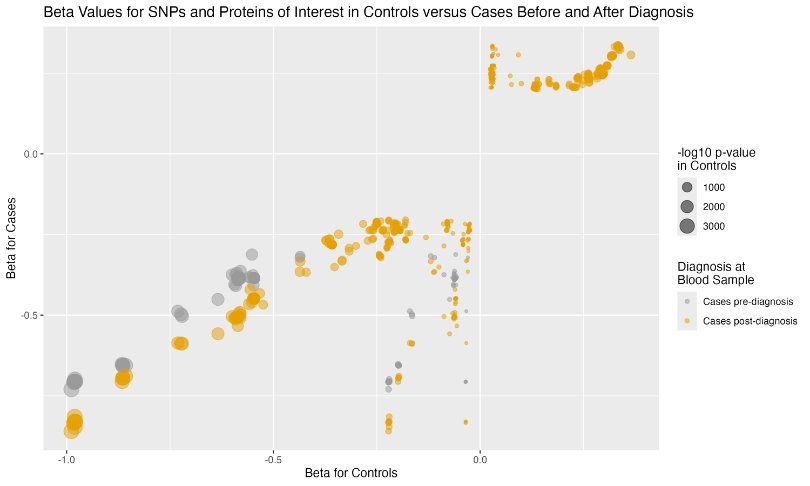


**Fig.S13** Beta values for SNPs and proteins of interest in control versus cases before and after diagnosis​

Graph comparing the Beta values generated in pQTL via linear regression between SNPs and protein quantity for TFF3, TFF1, SELE, RETN, LCN2, AHCY, CEACAM5. The cohort is split into controls who have never had colorectal cancer and cases who either have a diagnosis at the time of giving a blood sample or who will go on to receive a diagnosis after the blood sample. A filter has been applied so that both cases and controls must have generated a p-value for the SNP of <1e-5 to be included in this chart.


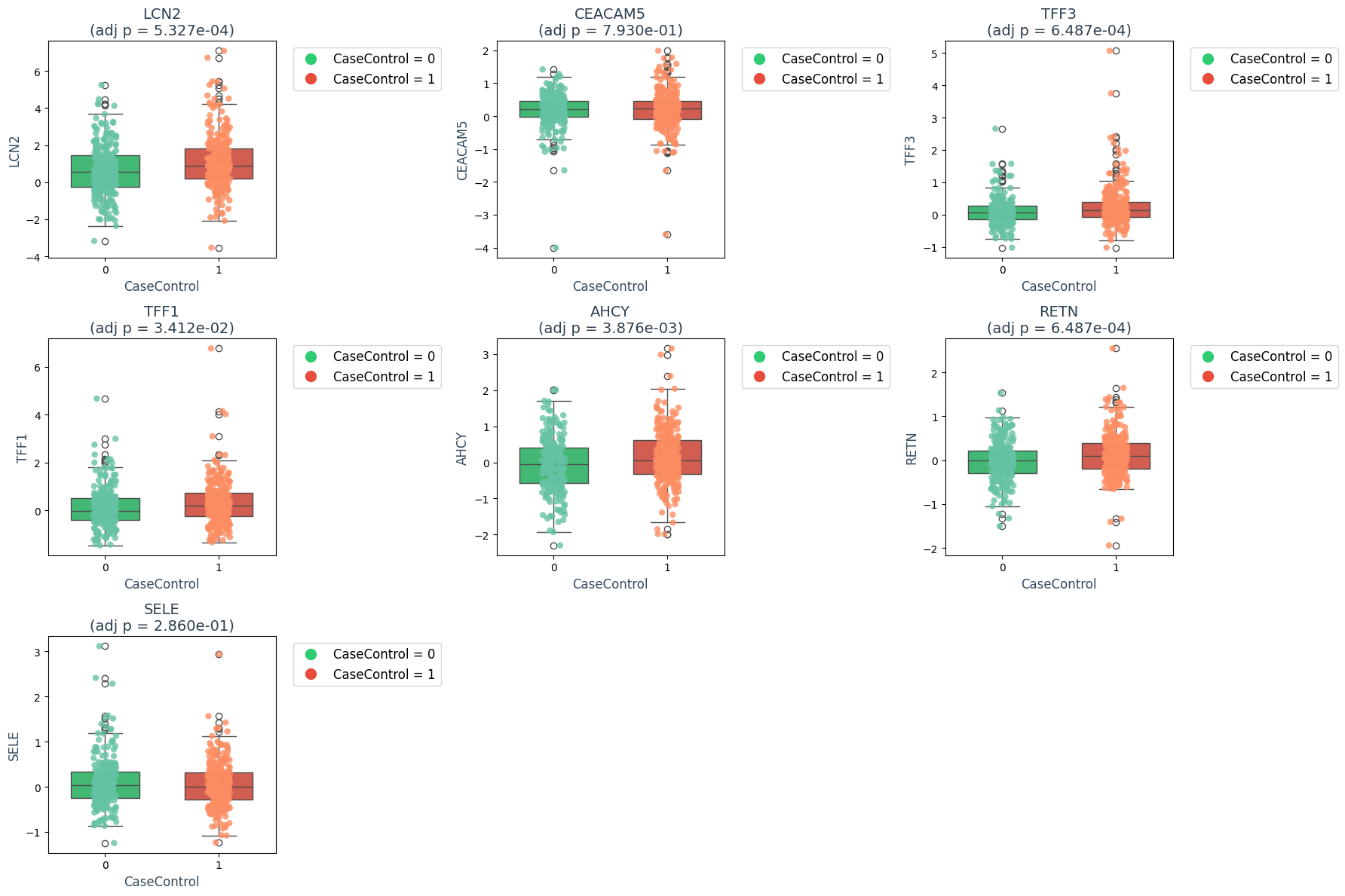


**Fig. S14** The distribution of the potential biomarker proteins in ComBat corrected dataset.


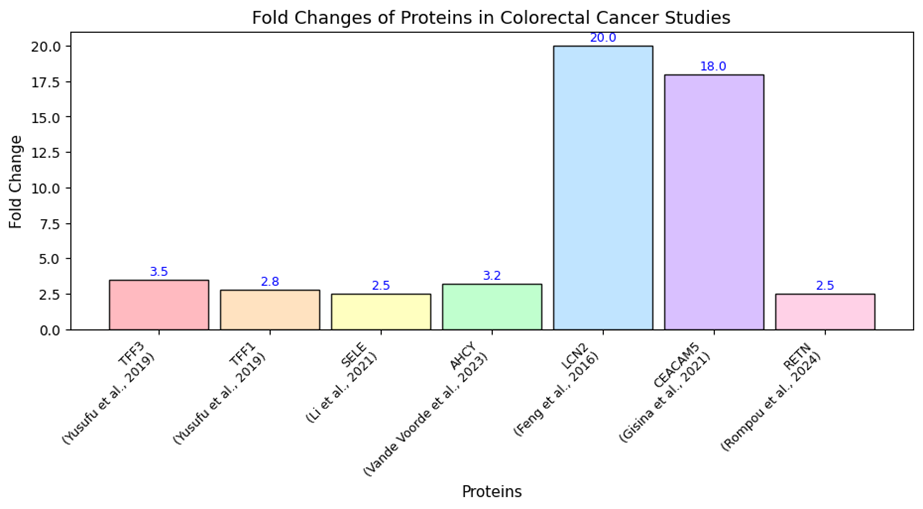


**Fig. S15** Fold changes of proteins in other colorectal cancer studies.
